# Considering New Regularization Parameter-Choice Techniques for the Tikhonov Method to Improve the Accuracy of Electrocardiographic Imaging

**DOI:** 10.3389/fphys.2019.00273

**Published:** 2019-03-27

**Authors:** Judit Chamorro-Servent, Rémi Dubois, Yves Coudière

**Affiliations:** ^1^IHU-Liryc, Electrophysiology and Heart Modeling Institute, Foundation Bordeaux Université, Bordeaux, France; ^2^CARMEN Research Team, INRIA, Bordeaux, France; ^3^Univ. Bordeaux, IMB UMR 5251, CNRS, Talence, France; ^4^Univ. Pompeu Fabra, PhySense Group, DTIC and BCN-Medtech, Barcelona, Spain; ^5^Univ. Bordeaux, Centre de Recherche Cardio-Thoracique de Bordeaux, U1045, Bordeaux, France; ^6^INSERM, Centre de Recherche Cardio-Thoracique de Bordeaux, U1045, Bordeaux, France

**Keywords:** inverse problem, Tikhonov, regularization, electrocardiography, MFS, ill-posed, ECG, body surface potentials

## Abstract

The electrocardiographic imaging (ECGI) inverse problem highly relies on adding constraints, a process called regularization, as the problem is ill-posed. When there are no prior information provided about the unknown epicardial potentials, the Tikhonov regularization method seems to be the most commonly used technique. In the Tikhonov approach the weight of the constraints is determined by the regularization parameter. However, the regularization parameter is problem and data dependent, meaning that different numerical models or different clinical data may require different regularization parameters. Then, we need to have as many regularization parameter-choice methods as techniques to validate them. In this work, we addressed this issue by showing that the Discrete Picard Condition (DPC) can guide a good regularization parameter choice for the two-norm Tikhonov method. We also studied the feasibility of two techniques: The U-curve method (not yet used in the cardiac field) and a novel automatic method, called ADPC due its basis on the DPC. Both techniques were tested with simulated and experimental data when using the method of fundamental solutions as a numerical model. Their efficacy was compared with the efficacy of two widely used techniques in the literature, the L-curve and the CRESO methods. These solutions showed the feasibility of the new techniques in the cardiac setting, an improvement of the morphology of the reconstructed epicardial potentials, and in most of the cases of their amplitude.

## Introduction

Cardiovascular diseases causes 17.9 million deaths every year, accounting for 31% of all global deaths. Electrocardiographic imaging (ECGI) is a non-invasive technique that reconstructs epicardial potentials and epicardial activation maps by combining body surface measurements with respective epicardial and body geometries. In a recent manuscript comparing the non-invasive ECGI with prior invasive techniques (Duchateau et al., 2018), the authors summarized the use of ECGI in different pre-clinical and clinical settings. While (Duchateau et al., 2018) highlights the favorable outcome of ECGI for treatment response prediction of cardiac resynchronization and ablation guidance for atrial fibrillation and ventricular tachycardia; it also states the need of further work on the ECGI inverse problem to improve its accuracy.

The ECGI inverse problem of computing epicardial potentials, Φ_*E*_, from the body surface measured potentials, Φ_*T*_, (MacLeod and Brooks, 1998; Ramanathan et al., 2004; Oostendorp et al., 2011; Wang et al., 2011; Oosterom van, 2012; Haissaguerre et al., 2013; Rudy, 2013; Cochet et al., 2014; Dubois et al., 2015; Shah, 2015) is an ill-posed problem (MacLeod and Brooks, 1998; Milanič et al., 2014; Cluitmans et al., 2015; Shah, 2015; Figuera et al., 2016). By introducing additional information, by using regularization techniques, we can overcome this ill-posedness (MacLeod and Brooks, 1998; Milanič et al., 2014; Cluitmans et al., 2015; Shah, 2015; Figuera et al., 2016).

Two recent manuscripts (Milanič et al., 2014; Figuera et al., 2016) studied the performance of different regularization techniques and concluded that due to the little differences among the more than 13 techniques used in each study, the most likely method to solve the ECGI problem in absence of prior information about the epicardial potentials was the two-norm Tikhonov regularization technique.

The two-norm Tikhonov regularization method (from now on referred to as Tikhonov) constrains the solution to be smooth or to have a small signal energy resolution. The Tikhonov regularization parameter weights the residual norm against the solution norm. Its role is to find a balance between solutions based on the body surface potential measurements and solutions that are constrained too much. Parameter-choice methods therefore became very data dependent (Hansen, 2010). Finally, regularization parameters that may perform well for a determined numerical model, may perform poorly when changing key factors of the model, such as the discretization or the boundary conditions (Hansen, 2010; Chamorro-Servent et al., 2016a,b). Then, for solving different clinical problems (different data) and different numerical models, it is preferable to have several automatic parameter-choice algorithms available (Hansen, 2010; Chamorro-Servent et al., 2016a,b).

In many cases, the regularization parameter, α, from the Tikhonov method is selected manually. This is done by subjectively choosing the value that provides the best results from a sequence of regularization parameters. The procedure becomes user dependent and time consuming and less likely reproducible. Several automatic methods have been suggested to overcome this problem. These include: (i) Strategies requiring prior knowledge of the noise (such as unbiased predictive risk estimator method, the discrepancy principle method, or the normalized cumulative periodogram), and (ii) strategies that do not need *a priori* information (such as zero-crossing method, Composite Residual and Smoothing Operator, L-curve, generalized cross-validation) (Hansen, 2010). For the ECGI, we will focus on the latter. In addition, from this latter group, we will focus on regularization parameter-choice methods that can easily be extended to the new goals (i.e., methods that not only consider information about the residual norm but also about the solution norm). This choice is due to the recent interest in improving the ECGI inverse solution by introducing physiological-based prior information on the regularization term (Figuera et al., 2016; Duchateau et al., 2018).

The automatic regularization parameter-choice method previously used in the ECGI literature, when using the method of fundamental solution (MFS) (Rudy, 2004; Wang and Rudy, 2006), without prior information, is the Composite Residual and Smoothing Operator (CRESO) technique (Colli-Franzone et al., 1985). The CRESO method has been found to provide the minimum root-mean-square error (RMSE) between the computed epicardial potentials (Φ_*E*_) and the measured ones (Rudy, 2004). When other numerical models were used to solve the ECGI problem (such as the Boundary Element method), the community has commonly used the L-curve method to find the regularization parameter (Milanič et al., 2014; Cluitmans et al., 2015; Figuera et al., 2016).

Both the CRESO and the L-curve methods have shown efficacy in the wide inverse problems' bibliography (Ruan et al., 1999; Rudy, 2004; Wang and Rudy, 2006; Hansen, 2010; Milanič et al., 2014; Cluitmans et al., 2015; Figuera et al., 2016). However, it becomes challenging to find an automatic regularization parameter-choice method for Tikhonov regularization that is suitable for all ill-posed inverse problems (Hansen, 2010). The CRESO and the L-curve techniques may require *a priori* information and/or manual adjustment (Rudy et al., 2006), due to an over-regularization of the solution. In addition, the convergence of the L-curve has failed in some cases, when the generalized Fourier coefficients of the data decayed at the same rate or a lower rate than the singular values (SVs) of the operator (Vogel, 1996).

PC Hansen showed that a necessary mathematical condition for the existence of a meaningful solution for Tikhonov regularization is the Discrete Picard Condition (DPC) (Hansen, 1990, 2010). DPC says exactly that “a good regularization parameter avoids SVs decaying to zero faster than the respective Fourier coefficients of the data.” The DPC has been used as a visual verification tool when studying the suitability of a regularization parameter for Tikhonov in several fields (Hansen, 1990, 2010; Chamorro-Servent et al., 2011), including ECG (Greensite et al., 1998). However, to the best of our knowledge an automatic DPC-based method does not exist yet.

Finally, the U-curve method has been introduced to overcome some drawbacks caused by the L-curve method in other fields (Krawzyck-Stando and Rudnicki, 2007; Chamorro-Servent et al., 2011; Chen et al., 2016), such as: (i) its non-convergence, (ii) the over smoothing of its solution, (iii) its lack of computational robustness when dealing with large scale problems. The L-curve computational cost has already been questioned in the ECGI setting (Figuera et al., 2016).

The target of this paper is to show the feasibility of the U-curve method, never used in the cardiac inverse problem setting, and to develop a new automatic DPC-based method, named ADPC. Both techniques are validated when using the MFS with simulated and experimental data. Their efficacy (in terms of amplitude and morphology preservation of the reconstructed potentials and dV/dT patterns) is compared with the existent L-curve and CRESO methods.

As a first step, we present the MFS and the Tikhonov regularization method, we summarize the role of the DPC in the Tikhonov regularization, and we introduce the different regularization parameter-choice methods. Afterwards, we describe the *in-silico* and experimental data, as well as the statistical analysis performed to compare the results. Later, we summarize the main results obtained. Finally, we draw conclusions and discuss the issues and the limitations raised.

## Methods

### The Method of Fundamental Solution (MFS) and l2-Norm Tikhonov Regularization

In the MFS (Wang and Rudy, 2006), the potential expression is defined as a linear combination of the Laplace fundamental solutions over a discrete set of virtual source points. The necessary virtual source points are located outside of Ω, where Ω is the domain of interest, specifically the volume conductor enclosed by the body surface (**Γ**_***T***_) and the epicardial surface (**Γ**_***E***_). The potential Φ for xϵΩ is stated as $\Phi \left(x\right)=a_{0}+\overset{N_{S}}{\underset{j=1}{\sum }}f\left(\left|x-y_{j}\right|\right)a_{j,}\;$ where the (***y***_***j***_)_***j*** = **1**..***N***_***S***__ are the ***N***_***S***_ fixed locations of the virtual sources points (***y***_***j***_ ∉ Ω), and the (***a***_***j***_)_***j*** = **1**..***N***_***S***__ are their respective coefficients. Here, ***f*** stands for the Laplace fundamental solution, $f\left(x,y_{j}\right)=\frac{1}{\text{4}\pi r}$, where ***r*** = |***x*** − ***y***_***j***_| is the 3D Euclidean distance. The ***N***_***S***_ = ***N***_***T***_ + ***N***_***E***_ virtual sources locations are fixed by deflating the ${\left(x_{i}^{E}\right)}_{i=1,2,\cdots \;,N_{E}}$ locations at **Γ**_***E***_ (by a numerical factor 0.8) and inflating the ${\left(x_{i}^{T}\right)}_{i=1,2,\cdots \;,N_{T}}$ electrodes locations at **Γ**_***T***_ (by a factor 1.2), relatively to the geometrical center of the heart. This deflation and inflation schemes are based on (Wang and Rudy, 2006).

The potentials on Γ_*E*_, Φ_*E*_ = $\left(\Phi \left(x_{i}^{E}\right)\right)$_*i* = 1, ··· ,*N*_*E*__, can be also expressed by the equation above as $\Phi \left(x_{i}^{E}\right)=a_{0}+\overset{N_{S}}{\underset{j=1}{\sum }}f\left(\left|x_{i}^{E}-y_{j}\right|\right)a_{j}$, where the only unknowns are the coefficients of the virtual sources (*a*_0_, *a*_1_, ··· , *a*_*N*_*S*__). Such coefficients are found in (Wang and Rudy, 2006) by imposing on Γ_*T*_ the Dirichlet (Φ = Φ_*T*_) and the zero-flux or homogeneous Neumann (∂_*n*_ Φ = 0) boundary conditions in an equivalent weight. This is done by using potential definition and the values of its normal derivatives, and it yields to solve the linear system

$$\begin{array}{l} \;\Phi \left(x_{i}^{T}\right)=a_{0}+\sum_{j=1}^{N_{S}}f\left({|x}_{i}^{T}-y_{j}|\right)a_{j}\;=\Phi_{T},\\ \partial_{n}\Phi \left(x_{i}^{T}\right)=a_{0}+\sum_{j=1}^{N_{S}}\partial_{n_{i}}f\left({|x}_{i}^{T}-y_{j}|\right)a_{j}\;=0 \end{array}$$

where Φ_*T*_ = (Φ_*i*_)_*i* = 1, ··· , *N*_*T*__ are the potentials recorded on the ${\left(x_{i}^{T}\right)}_{i=1,2,\cdots \;,N_{T}}$ torso electrodes locations.

This system can be written in a matrix notation as *Ma* = *b*, being

$$\begin{array}{l} M=\left(\begin{array}{c} \begin{array}{ccc} 1\; & \;f\left({|x}_{1}^{T}-y_{1}|\right)\;\cdots & f\left(|x_{1}^{T}-y_{N_{S}|}\right)\\ \;\vdots \; & \;\ddots & \vdots \\ 1\; & \;f\left({|x}_{N_{T}}^{T}-y_{1}|\right)\;\cdots & \;f\left(\left|x_{N_{T}}^{T}-y_{N_{S}}\right|\right) \end{array}\\ \begin{array}{ccc} \;0 & \partial_{n_{1}}f\left(\left|x_{1}^{T}-y_{1}\right|\right)\;\cdots & \partial_{n_{1}}f\left({|x}_{1}^{T}-y_{N_{S}}|\right)\\ \;\vdots & \;\ddots & \vdots \\ \;0 & \partial_{n_{N_{T}}}f\left(\left|x_{N_{T}}^{T}-y_{1}\right|\right)\;\cdots & \partial_{n_{N_{T}}}f\left({|x}_{N_{T}}^{T}-y_{N_{S}}|\right) \end{array}\\ \; \end{array}\right), \end{array}$$

$a={\left(a_{0},a_{1},{\cdots \;,a}_{N_{S}}\right)}^{T}\epsilon {\mathbb{R}}^{1+N_{s}}$ and $b=\left(\begin{array}{c} \Phi_{T}\\ 0\\ \; \end{array}\right)\epsilon {\mathbb{R}}^{2N_{T}}$.

Then, finding the sources coefficients ($a\epsilon {\mathbb{R}}^{1+N_{s}}$) results in solving a quadratic minimization problem

$$\begin{array}{l} J(a,\alpha )=\frac{1}{2}{\left\|Ma-b\right\|}^{2}+\frac{\alpha^{2}}{2}{\left\|a\right\|}^{2}, \end{array}$$

where α > 0 is the Tikhonov regularization parameter.

The Tikhonov solution can be defined in terms of singular values (SV) decomposition of *M* (*M* = *USV*^*T*^), by equaling the gradient of *J*(*a*, α) to zero and writing *I* = *VV*^*T*^

$$\begin{array}{l} {\nabla J}_{a}\left(a,\alpha \right)=\frac{1}{2}\nabla \left({\left(Ma-b\right)}^{T}\left(Ma-b\right)+\alpha^{2}Ia^{T}a\right)\\ =\left(M^{T}M\right)a-M^{T}b{+\alpha }^{2}Ia=\;0,\\ a_{\alpha }=\;{\left(M^{T}M+\alpha^{2}I\right)}^{-1}M^{T}b=\sum_{i=1}^{\min\left(2^{*}N_{T,}N_{S}+1\right)}\\ \frac{\sigma_{i}^{2}}{\sigma_{i}^{2}+\alpha^{2}}u_{i}^{T}bv_{i}=\sum_{i=1}^{\min\left(2^{*}N_{T,}N_{S}+1\right)}\frac{\sigma_{i}^{2}}{\sigma_{i}^{2}+\;\alpha^{2}}\frac{u_{i}^{T}b}{\sigma_{i}}v_{i}, \end{array}$$

where σ_*i*_ are the SVs (the elements of the diagonal matrix *S*) in descending order, $\sigma_{1}\geq \cdots \geq \sigma_{\min\left(2^{*}N_{T,}N_{S}+1\;\right)}$.

Once the Tikhonov regularization problem has been solved, we can calculate the epicardial potentials, $\Phi \left(x_{i}^{E}\;\right)$.

### Discrete Picard Condition (DPC)

The DPC is satisfied “if the so-called Fourier coefficients of the right-hand side (when expressed in terms of the generalized SV decomposition coefficients), $\left|u_{i}^{T}b\right|$, decay to zero faster than the respective generalized SVs, σ_*i*_'s.” In other words, the regularization parameter must be used to control the undesired high-frequency oscillations that contaminate the solution.

The Picard plot (Hansen, 1990, 2010), depicts the $\left|u_{i}^{T}b\right|$ and σ_*i*_-values against their respective quotient in a same logarithmic scale plot.

In ill-posed problems the solution coefficients $\frac{\left|u_{i}^{T}b\right|}{\sigma_{i}}$ increase for larger values of the index *i*. Hence, the computed solutions ($a_{\alpha }=\overset{\min\left(2^{*}N_{T,}N_{S}+1\right)}{\underset{i=1}{\sum }}\frac{\sigma_{i}^{2}}{\sigma_{i}^{2}+\alpha^{2}}\frac{u_{i}^{T}b}{\sigma_{i}}v_{i}\;$above) are completely dominated by the smallest SVs. In these cases, if we want to calculate a satisfactory solution by means of Tikhonov regularization, the DPC must be fulfilled (Vogel, 1996; Hansen, 2010). The DPC allows to balance how well the regularized solution approaches the unknown (i.e., the exact solution). The σ_*i*_ above the regularization parameter α (useful SVs) must decay to zero less quickly than the corresponding right-hand side coefficients, $\left|\;u_{i}^{T}b\right|$. In other words, the DPC says that an ill-conditioned system must be regularized if a suitable solution is to be obtained and a solution based on a vector $\frac{\left|u_{i}^{T}b\right|}{\sigma_{i}}$ that only increases is generally not useful.

### Automatic Regularization Techniques

#### Composite Residual and Smoothing Operator (CRESO)

The CRESO method (Colli-Franzone et al., 1985) was presented as a practical method but has turned out to be extensively accepted as the preferred parameter choice-method in widely ill-posed bioelectric inverse problems (Ruan et al., 1999). It chooses the parameter value which produces the first local maximum of the difference between the derivative of the regularization term and the derivative of the residual term

$$\begin{array}{l} C(\alpha )=\left\{\frac{d}{d(\alpha^{2})}(\alpha^{2}{\left\|a(\alpha )\right\|}^{2})-\frac{d}{d(\alpha^{2})}{\left\|Ma(\alpha )-b\right\|}^{2},\;\alpha >0\right\} \end{array}$$

#### L-Curve

The L-curve has become the best-known method for assessing a regularization parameter-value in widely ill-posed problems fields (Hansen, 1990; Hansen and O'Leary, 1993; Ruan et al., 1999). It is defined in terms of

$$\begin{array}{l} L(\alpha )=\left\{\left(\left\|Ma(\alpha )-b\right\|,\left\|a(\alpha )\right\|\right),\;\alpha >0\right\} \end{array}$$

If we plot the L-curve, it has a L-shape and we can choose the regularization parameter value by using Hansen and O'Leary's criterion (Hansen, 1990; Hansen and O'Leary, 1993). This criterion chooses the α-value corresponding to the point of maximum curvature on the log-log plot of the L-curve.

#### U-Curve

The U-curve (Krawzyck-Stando and Rudnicki, 2007) is defined as the log-log scale plot of the sum of the inverse of the regularized solution norm ‖*a*(α)‖ and the respective residual error norm ‖*Ma*(α) − *b*‖, for α > 0

$$\begin{array}{l} U\left(\alpha \right)=\frac{1}{||Ma\left(\alpha \right)-b|{|}^{2}}+\frac{1}{||a\left(\alpha \right)|{|}^{2}}\; \end{array}$$

The U-curve plot has a U-shape. The optimum regularization parameter is the value for which the U-curve achieves its minimum. And the sides of the U-curve correspond to the regularization values for which either the solution norm or the residual norm dominates. When dealing with large scale problems, the U-curve is computationally efficient. This is due to its a priori interval definition where the appropriate regularization parameter is located (Krawzyck-Stando and Rudnicki, 2007; Chamorro-Servent et al., 2011; Chen et al., 2016).

#### ADPC: A New Regularization Parameter Choice Method

As mentioned previously, an optimal regularization value, α, for Tikhonov method, when dealing with l2-norm constraints, must fulfill the DPC (Hansen, 1990, 2010). This means that the σ_*i*_ above the suitable α must not decay to zero faster than the corresponding $\left|u_{i}^{T}b\right|$, to avoid the computed Tikhonov solutions (*a*_α_) from being entirely dominated by the smallest SVs.

**Figure 1:**
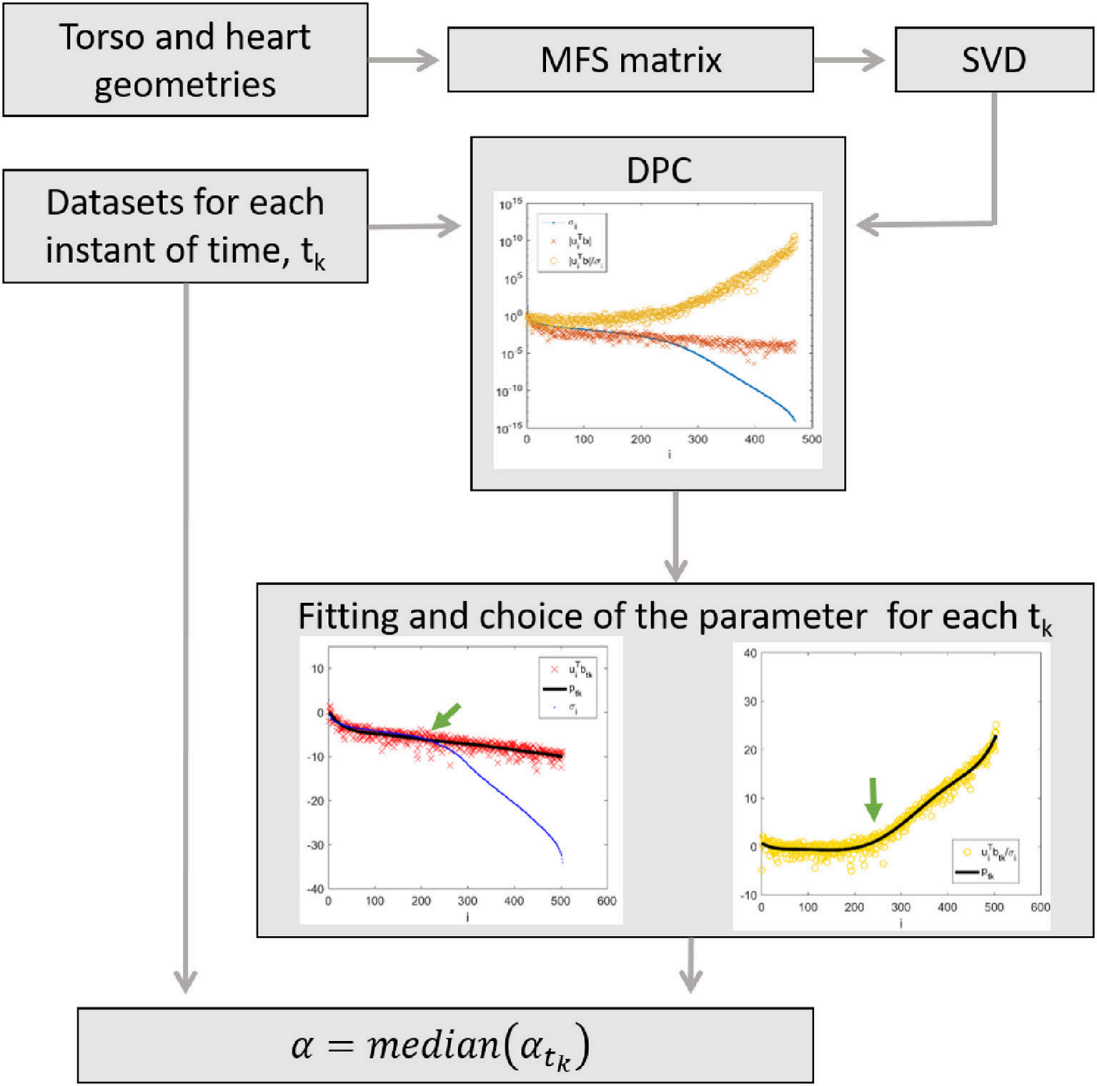
Flowchart illustrating the algorithm.

Based on the DPC, we performed an automatic regularization parameter-choice algorithm (Figure 1):

1. We computed the SV decomposition of the MFS matrix, *M*, to find the SVs (σ_*i*_) and the left singular vectors (*u*_*i*_).
2. For each time step, *t*_*k*_(*ms*), we calculated the log$\left(|u_{i}^{T}b_{t_{k}}|\;\right)$ and log$\left(|u_{i}^{T}b_{t_{k}}|\;/{\;\sigma }_{i}\right)$ and we fit both of them by two polynomials ${p\left(i,\log\left(\left|u_{i}^{T}b_{t_{k}}\right|\right)\;\right)}_{t_{k}}$ and ${q\left(i,\log|u_{i}^{T}b_{t_{k}}|\;/{\;\sigma }_{i}\;\right)}_{t_{k}}$ of degree from 5 to 7, where *k* = 1, ··· , *N*_*t*_ are the time instants. Hence, we obtained: *p*_*t*_1_, ··· _,*p*_*t*__*N*_*t*____ and *q*_*t*_1_, ··· _,*q*_*t*__*N*_*t*____, two polynomials set for each time step *t*_*k*_.
3. For each pair of polynomials at each time step, *t*_*k*_, we found: α_*t*_*k*__ = σ_max{*i*}_ (σ_0_ ≥ σ_1_ ≥ ⋯ ≥ σ_*r*_ > 0), such that DPC was fulfilled.
4. The suitable ADPC regularization parameter was defined as: α = median(α_*t*_*k*__).

Steps two and three of this algorithm consists in the lower limit that any suitable Tikhonov regularization value can attain to still fulfill the DPC. Step three consists of looking for the index *i*, which corresponds to the last SV, before the small SVs coefficients start to dominate the solution. That means, previously log(σ_*i*_) starts to decrease faster than log$\left(|u_{i}^{T}b_{t_{k}}|\;\right)$. The fitting of the log$\left(|u_{i}^{T}b_{t_{k}}|\;\right)$ and log$\left(|u_{i}^{T}b_{t_{k}}|\;/{\;\sigma }_{i}\right)$ by two polynomials in step two is done to simplify the automatic achievement of the optimal index *i* (in step three).

### *In-silico* and Experimental Data

A total of sixteen datasets were used to test our algorithms, eight *in-silico* data and eight experimental data. In both cases, body surface potentials and epicardial potentials were provided.

#### *In-silico* Data

**Figure 2:**
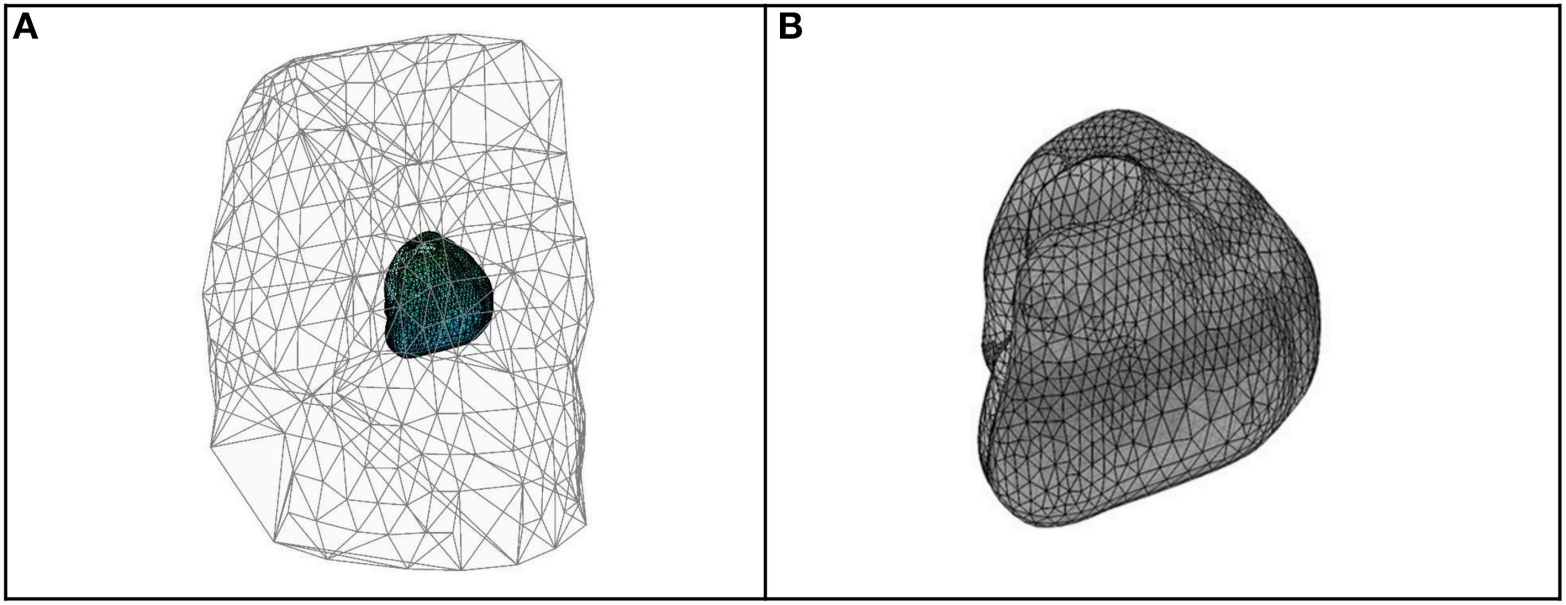
Geometries and meshes of the *in-silico* used data. **(A)** Body surface and heart geometries, and body surface mesh. **(B)** Heart geometry and mesh.

To test the effect of the new approaches described and to compare them with previous ones, eight *in-silico* different activation patterns were used (Duchateau et al., 2017). This included, one single site pacing in the right ventricular free wall, three single sites pacing in the left ventricular (lateral endocardial wall, mid wall, and lateral epi) and four single spiral waves. A monodomain reaction-diffusion model was simulated in a realistic 3D model of the human ventricles to mimic the propagating activation (Duchateau et al., 2017). The Ten Tusscher et al. model for the human ventricular myocyte (Ten Tusscher et al., 2004) was used to compute the transmembrane ionic currents. These currents were used to calculate the extracellular potential distribution all over the torso, by solving a static bidomain problem in a torso mode at 1 mm resolution (Potse et al., 2009). The torso model had heterogeneous conductivity, with anisotropic skeletal muscle, lungs, and intracavitary blood. The heart model comprised of right and left ventricles at 0.2 mm spatial resolution. From the rule-based fiber orientation derived an anisotropic conduction in the heart model. Both, heart and thoracic anatomies were based on MRI data (Figure 2). *In-silico* Φ_*T*_ and Φ_*E*_ every 1 ms were provided by these simulations.

#### Experimental Data

To test how much the regularization parameter-choice depended on the datasets chosen and to facilitate later comparison with other possible algorithms, we decided to use, in addition to the simulated data, eight datasets from the Experimental Data and Geometric Analysis Repository (EDGAR) (Aras et al., 2015) hosted by the SCI Institute at the University of Utah and freely distributed. The purpose of EDGAR is to share and collate electrocardiological data, specifically for the validation and advancement of ECGI problems among a worldwide consortium of academic institutions.

In the EDGAR data used, both potentials from the body surface and epicardial were simultaneously measured. The data selected for this study was: Sinus rhythm and paced beats from (i) a canine experiment (paced from the epicardial left ventricular apex) (Aras et al., 2015; Cluitmans et al., 2017) and (ii) from a pig experiment (Aras et al., 2015; Bear et al., 2015). And a control and three myocardial ischemia from a canine experiment, where the high right atrium was paced while an occlusion to the LAD induced ischemia (Aras et al., 2015).

In (Cluitmans et al., 2017), a computed tomography scan was first performed to localize the electrodes and epicardial surface, second, the body-surface potentials were recorded with 192 electrodes simultaneously to 67 electrodes implanted around the epicardium via a thoracotomy. In Bear et al. (2015) epicardial electrodes were placed with a custom-made elastic sock containing 239 unipolar silver-wire electrodes (5-to 10-mm spacing) drawn over the ventricles, after which the thorax was closed, and air expelled. Flexible strips (BioSemi, Amsterdam, The Netherlands) containing 184 electrodes (30- to 45-mm spacing) were attached to the body surface. Epicardial and body surface potentials were bandlimited (0.05–1,000 Hz) and recorded simultaneously at 2 kHz using separate acquisition systems (UnEmap, Auckland Uniservices Ltd, Auckland, New Zealand and ActiveTwo, BioSemi, respectively). Magnetic resonance imaging from the heart and thorax were acquired by placing contrast markers on the sock and body surface strips to localize the electrodes. Finally, the signals were temporally aligned by identifying the onset of a short burst of square 2 ms pulses recorded simultaneously on a single channel in both the systems.

### Statistical Analysis

We computed the potentials on the epicardium for the diverse regularization parameters choices.

Afterwards, correlation coefficients (CCs) and relative root-mean squared errors (rRMSEs) were computed over the time steps as specified below.

$$\begin{array}{lll} CC & = & \frac{\sum_{i=1}^{N_{L^{*}}}\left(\Phi_{TE_{i}}-\;\bar{\Phi_{TE_{i}}}\right)\left(\Phi_{CE_{i}}-\;\bar{\Phi_{CE_{i}}}\right)}{\sqrt{\sum_{i=1}^{N_{L^{*}}}{\left(\Phi_{TE_{i}}-\;\bar{\Phi_{TE_{i}}}\right)}^{2}}\;\sqrt{\sum_{i=1}^{N_{L^{*}}}{\left(\Phi_{ME_{i}}-\;\bar{\Phi_{ME_{i}}}\right)}^{2}}}\;\\ rRMSE & = & \sqrt{\frac{\sum_{i=1}^{N_{L^{*}}}{\left({\Phi_{CE_{i}}-\Phi }_{TE_{i}}\right)}^{2}}{\sum_{i=1}^{N_{L^{*}}}{\left(\Phi_{TE_{i}}\right)}^{2}}}\; \end{array}$$

where Φ_*TE*_ were the target potentials and Φ_*CE*_ the computed ones. For the *in-silico* data, the Φ_*TE*_ were the simulated epicardial potentials and the Φ_*CE*_ the reconstructed ones at the same ${N_{L^{*}}=N}_{LE}$ locations. In the case of the experimental data, the Φ_*TE*_ were the potentials measured on the heart, and Φ_*CE*_ the reconstructed at the $N_{L^{*}}=N_{LS}$ closest epicardial locations.

Lastly, we showed the respective boxplots to allow their comparison. For the *in-silico* data, we also computed the dV/dT patterns and the correspondent correlation coefficients and the relative root-mean squared errors. We showed them in a table in the format [Median, (min, max)]. The highest correlation coefficients (CC) represents the best morphology and the lowest relative root mean-square error (rRMSE) represents the best amplitude of the reconstructed potentials.

## Results

### *In-silico* Data

**Figure 3:**
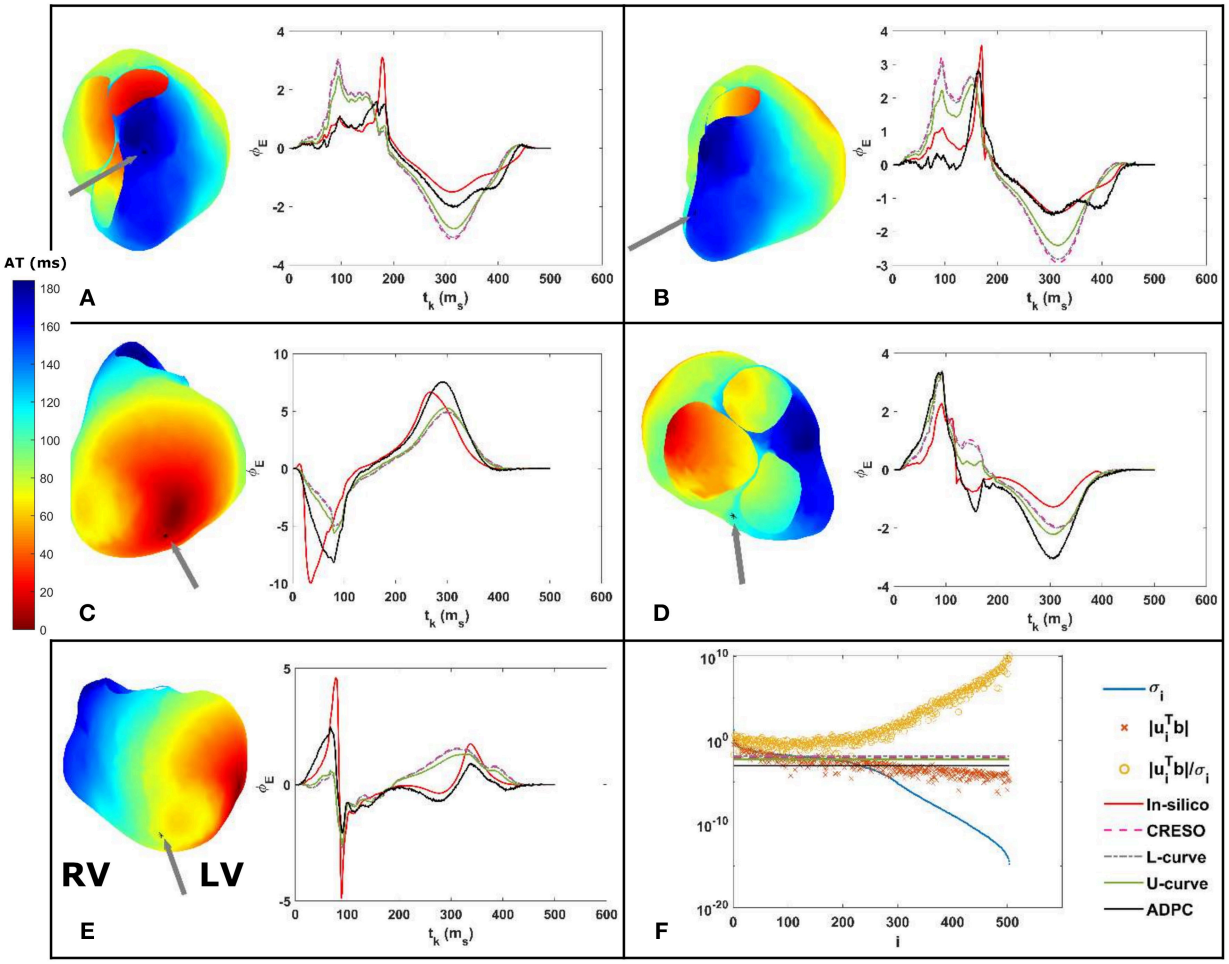
**(A–E)** On the right: reconstructed potentials provided by the different regularization parameters against the *in-silico* heart potentials. On the left: arrows on the 3D *in-silico*/reference AT map correspond to the spatial heart locations where each respective potential is shown. The arrow on the 3D map of figure **(C)** shows the single site pacing in the left ventricle lateral midwall. **(F)** DPC plot for t_k_ = 100 ms with the different computed regularization parameter values drawn as horizontal lines. The legend included in **(F)** serves the respective DPC plot, as well as the **(A–E)** potential plots discussed in this figure.

**Figure 4:**
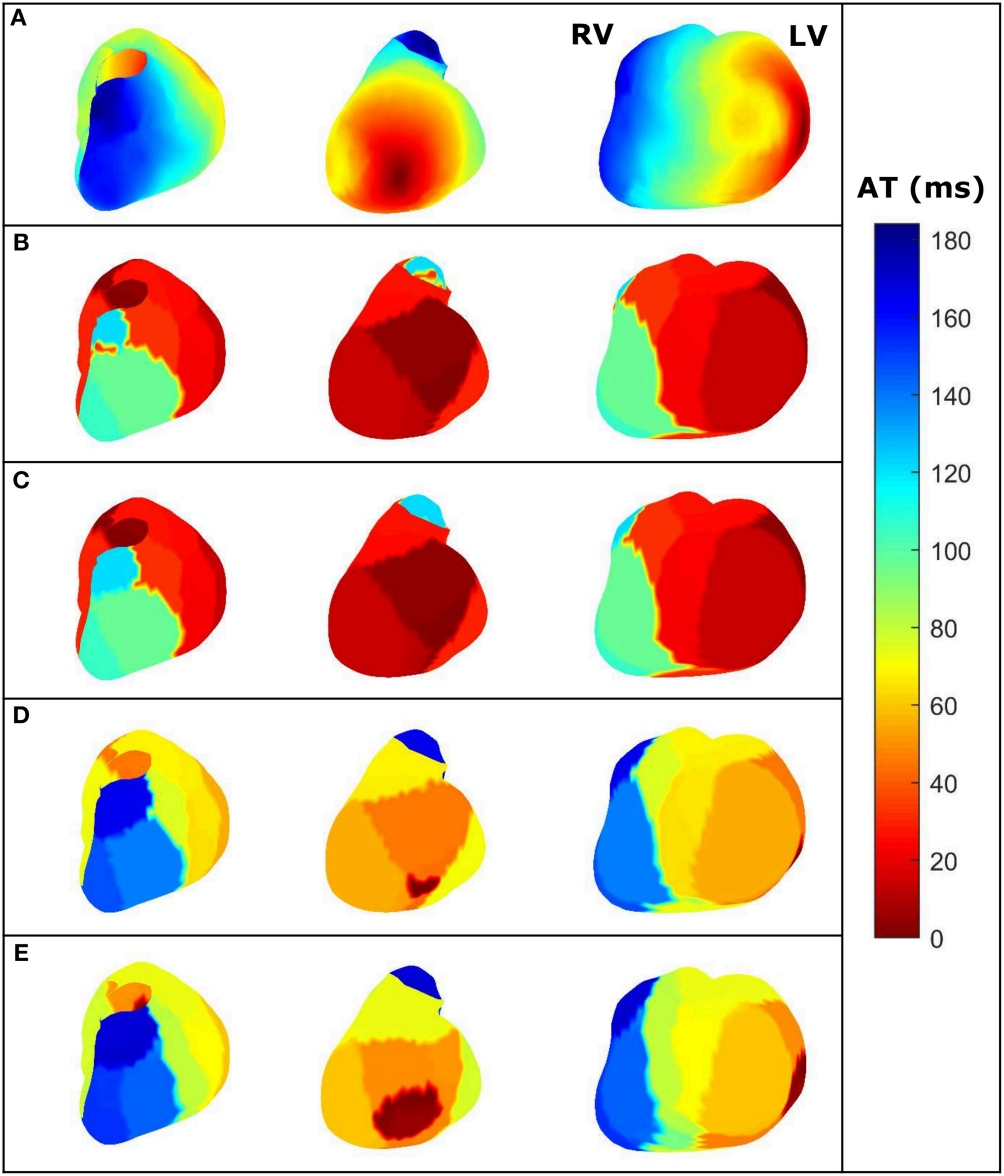
For the same dataset employed in Figure 3—three different views of the AT maps reconstructed from the heart potentials from: **(A)** *In-silico* reference data, **(B)** CRESO solution, **(C)** L-curve solution, **(D)** U-curve solution, and **(E)** ADPC solution. RV and LV are denoted in the *in-silico* AT map for reference.

Some of the effects related to the regularization parameter choice methods for a single site pacing in the midwall left ventricle *in-silico* dataset are depicted in Figures 3, 4 below. The reconstructed potentials by the different regularization parameter-choice methods are plotted against the *in-silico* heart potentials in Figures 3A–E. Next to each subplot an arrow marks, on the heart geometry, the location where the potentials are shown. In addition, Figures 3F shows the DPC plot depicting the resulting regularization parameter values on horizontal lines.

The activation time (AT) maps for the single site pacing in the midwall left ventricle *in-silico* dataset are shown in Figure 4.

**Figure 5:**
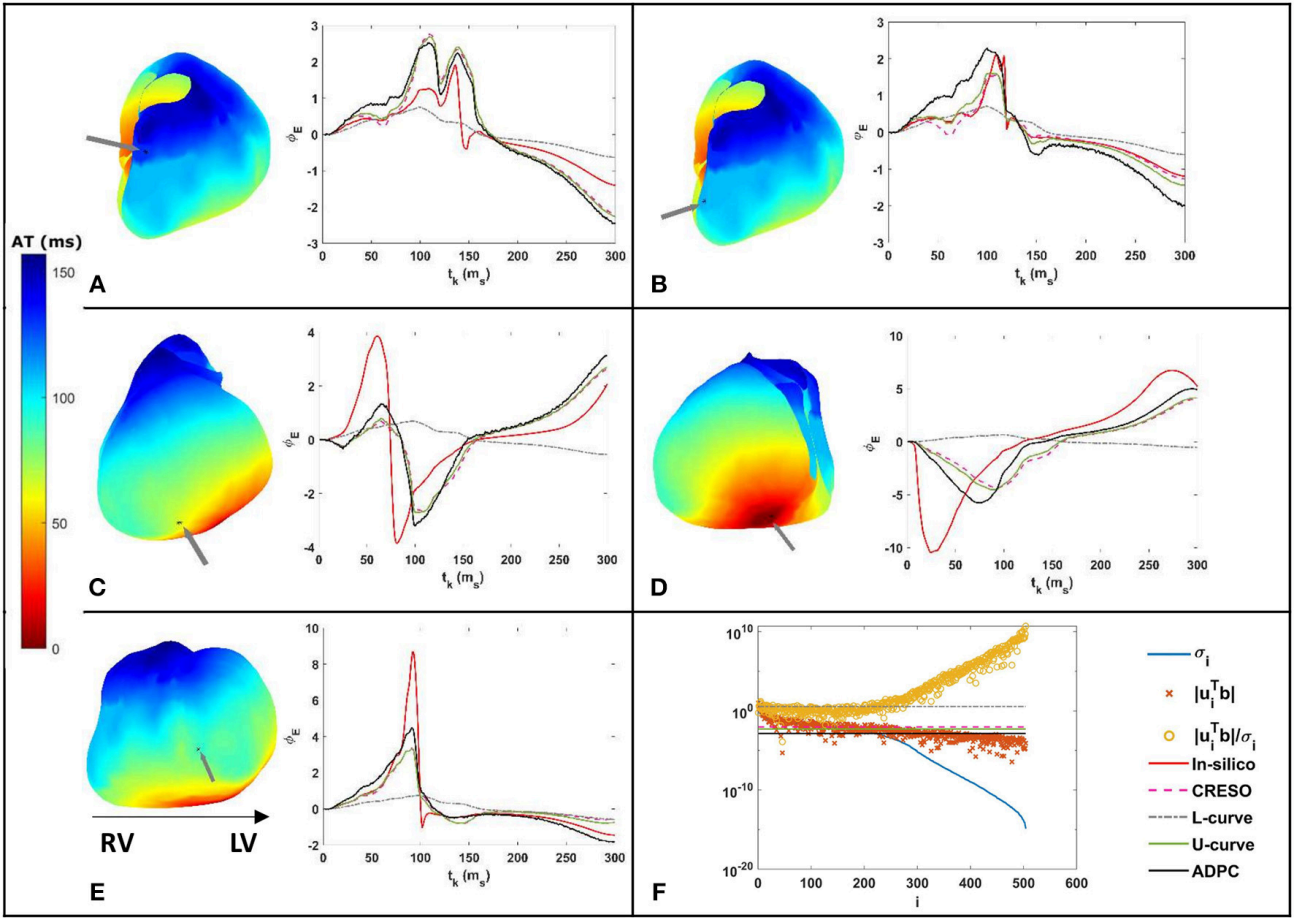
**(A–E)** On the right: reconstructed potentials provided by the different regularization parameters against the *in-silico* heart potentials. On the left: arrows on the 3D *in-silico*/reference AT map correspond to the spatial heart locations where each respective potential is shown. **(F)** DPC plot for t_k_ = 100 ms with the different computed regularization parameters values drawn as horizontal lines. The legend included in **(F)** serves the respective DPC plot, as well as the **(A–E)** potential plots discussed in this figure.

**Figure 6:**
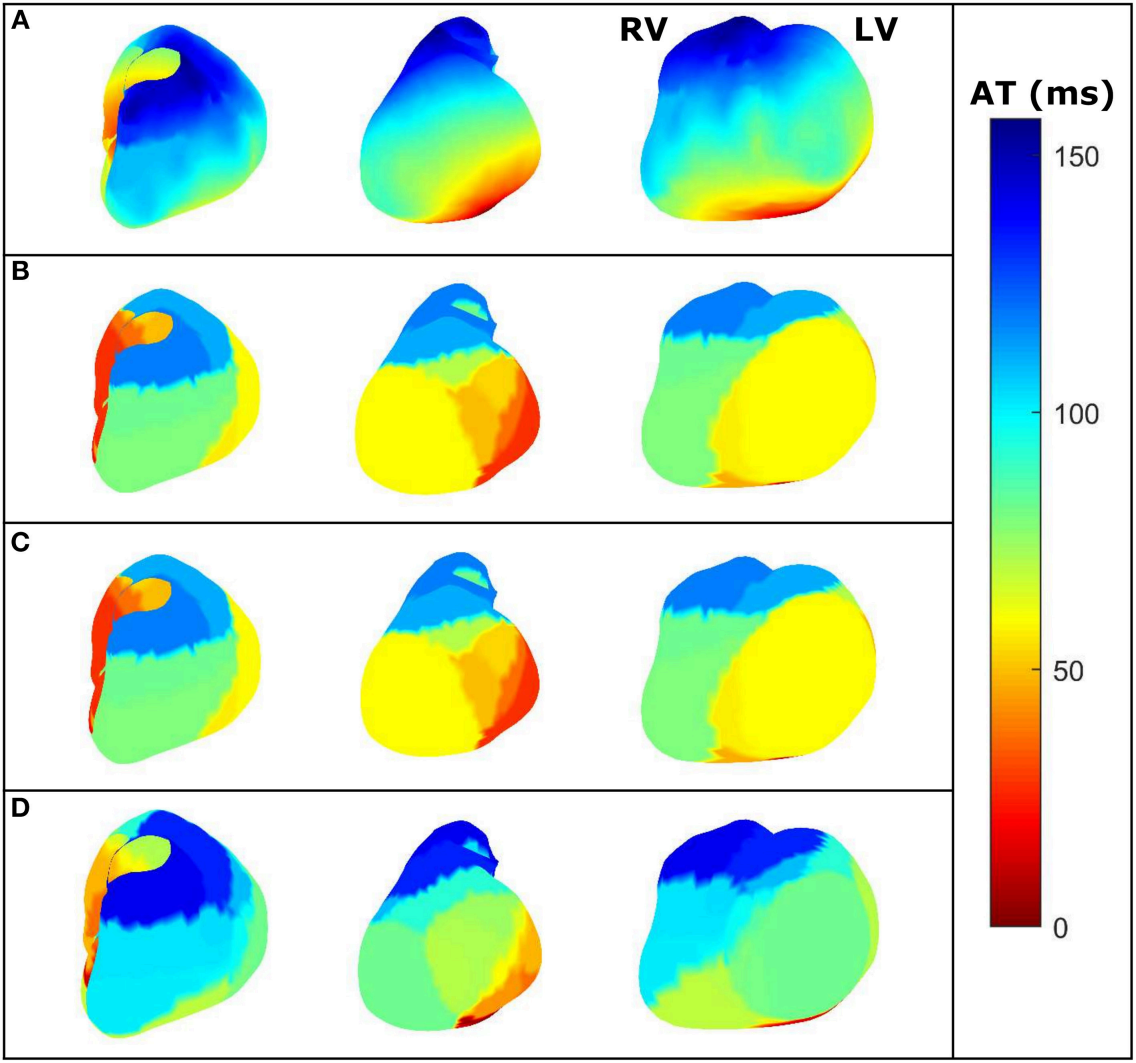
For the same dataset employed in Figure 5—three different views of the dV/dT maps reconstructed from the heart potentials from: **(A)** *in-silico* reference data, **(B)** CRESO solution, **(C)** U-curve solution and **(D)** ADPC solution. The L-curve inhibited the dV/dT map computation. RV and LV are denoted in the *in-silico* AT map for reference.

Similarly, Figures 5, 6 depict the same results for a single spiral wave with increased transverse conductivity. In addition, in the Supplementary Material, we included the dV/dT maps of the six additional *in-silico* datasets.

We can see on both DPC plots (Figures 3F, 5F) that the ADPC regularization parameter was chosen just before the SVs (σ_*i*_) start to decay faster than the respective $\left|u_{i}^{T}b_{t_{k}}\right|$. That moment corresponds to the moment just before $|u_{i}^{T}b_{t_{k}}|/\sigma_{i}\;$starts to increase fast. If we look at the values of the solution vectors, $|u_{i}^{T}b_{t_{k}}|/\sigma_{i}$, and we try to find a minimum, followed by a significant growth in the moving average; the point where the average grows above the minimum by a certain factor, locate the points were the high frequencies starts dominating. It is well-known than when high frequencies dominate, any error, artifact, or noise will start to dominate the solution. Our regularization parameter must therefore be chosen just before this starts to happen.

**Figure 7:**
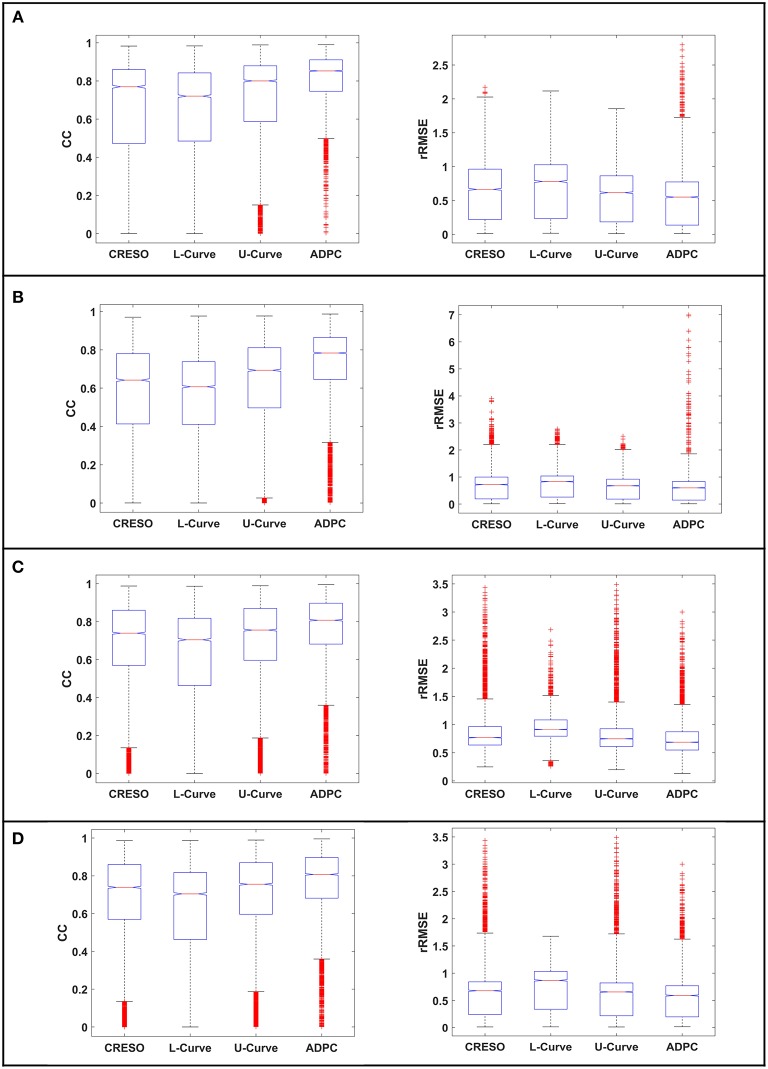
Boxplots of the correlation coefficients (CC) and the relative root-mean squared errors (rRMSE) between the reconstructed potentials and the respective *in-silico* heart potentials: **(A,B)** for the single site pacing simulations, **(C,D)** for the spiral simulations. **(A,C)** for all the 501-time steps where the heart data was simulated. **(B,D)** for the 200-time steps where we calculate the dV/dT maps. The red crosses denote the outlayers.

The statistics for the eight simulations datasets are shown on Figure 7 and Table 1. They compile the effect of the choice of the regularization parameter, on the reconstructed potentials (boxplots Figure 7) and on the dV/dT maps (Table 1). The relative root-mean squared errors give an estimate of the amplitude difference and the correlation coefficients give an estimate of the similarity of potential patterns or electrogram morphologies between measured and reconstructed data. We are interested in the highest correlation coefficients (best morphology) and the lowest relative root-mean squared errors (best amplitude). With the boxplots, we included statistics referring to all the 501-time steps where the heart potentials were simulated (Figures 7A,C) and the ones referring only to the 200-time steps where the dV/dT maps were reconstructed (Figures 7B,**D**). Finally, in the Supplementary Material, we included the boxplots of the reconstructed potentials for each individual dataset.

**Table 1:** Median [min, max]% differences of **(A–C)** the correlation coefficients and **(B–D)** the relative root-mean squared errors between each reconstructed dV/dT patterns and the dV/dT pattern resulting from the *in-silico* heart potentials.

**(A)**				**(B)**			
**CORRELATION COEFFICIENTS (CC)**				**RELATIVE ROOT-MEAN-SQUARED ERROR (rRMSE)**			
**CRESO**	**L-Curve**	**U-curve**	**ADPC**	**CRESO**	**L-Curve**	**U-curve**	**ADPC**
0.7735	NA^*^	**0.8343**	0.7948	0.5006	1	0.4272	**0.3780**
0.7691	NA^*^	0.8307	**0.9039**	0.5801	1	**0.5274**	**0.4395**
0.7817	0.8215	0.8498	**0.8961**	0.6063	0.5803	0.2419	**0.2009**
0.8224	0.8387	0.8702	**0.9091**	0.3248	0.3107	0.2915	**0.2665**
**(C)**				**(D)**			
**CORRELATION COEFFICIENTS (CC)**				**RELATIVE ROOT-MEAN-SQUARED ERROR (rRMSE)**			
**CRESO**	**L-Curve**	**U-curve**	**ADPC**	**CRESO**	**L-Curve**	**U-curve**	**ADPC**
0.8505	0.8317	0.8733	**0.9053**	0.3062	0.3187	0.2904	**0.2683**
0.8535	NA^*^	0.8704	**0.8902**	0.2019	1	**0.1977**	0.2134
0.8320	NA^*^	0.8531	**0.8971**	0.2717	1	0.1985	**0.1683**
0.8371	NA^*^	**0.8386**	0.8266	0.44	1	0.4654	**0.321**

(A,B) For the single site pacing. (C,D) From the in-silico heart potentials for the spirals. NA

* *Not applicable because the computation of the dV/dT patterns is inhibited due to the over-regularized solution provided by L-curve. The best results are highlighted in bold*.

The L-curve provided a clearly over-regularized solution for the singular single pacing in the right ventricle and for three of the single spiral waves inhibiting the computation of some of the dV/dT maps.

## Experimental data

**Figure 8:**
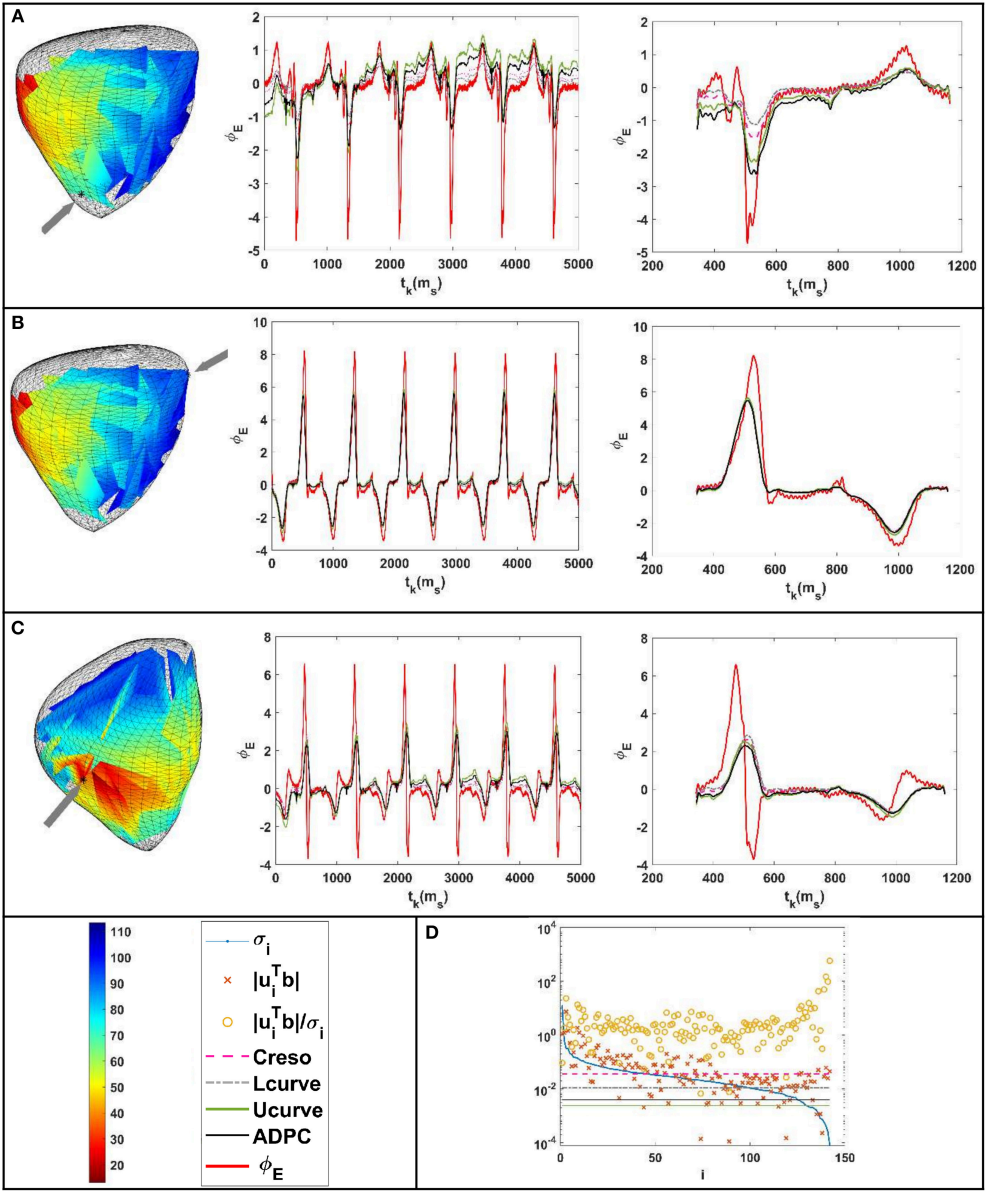
Results for the paced beat of the pig experiment (Bear et al., 2015). **(A–C)** From the left to the right: location of the epicardium where the potentials were compared (marked with an arrow above the recorded activation pattern). Reconstructed potentials against the measured ones for all the time steps and all the parameter-choice methods such as indicated in the legend below. Respective zoom (of the reconstructed potentials against the measured ones) at the t_k_ interval comprised between 343 and 1,161 ms. **(D)** DPC plot at t_k_ = 472 ms with the different regularization parameters values holding on horizontal lines following the legend.

Like Figures 3, 5, the Figure 8 shows the reconstructed potentials for the paced pig experiment referred to in section Experimental data against the measured potentials, and the DPC with the different regularization values chosen.

**Figure 9:**
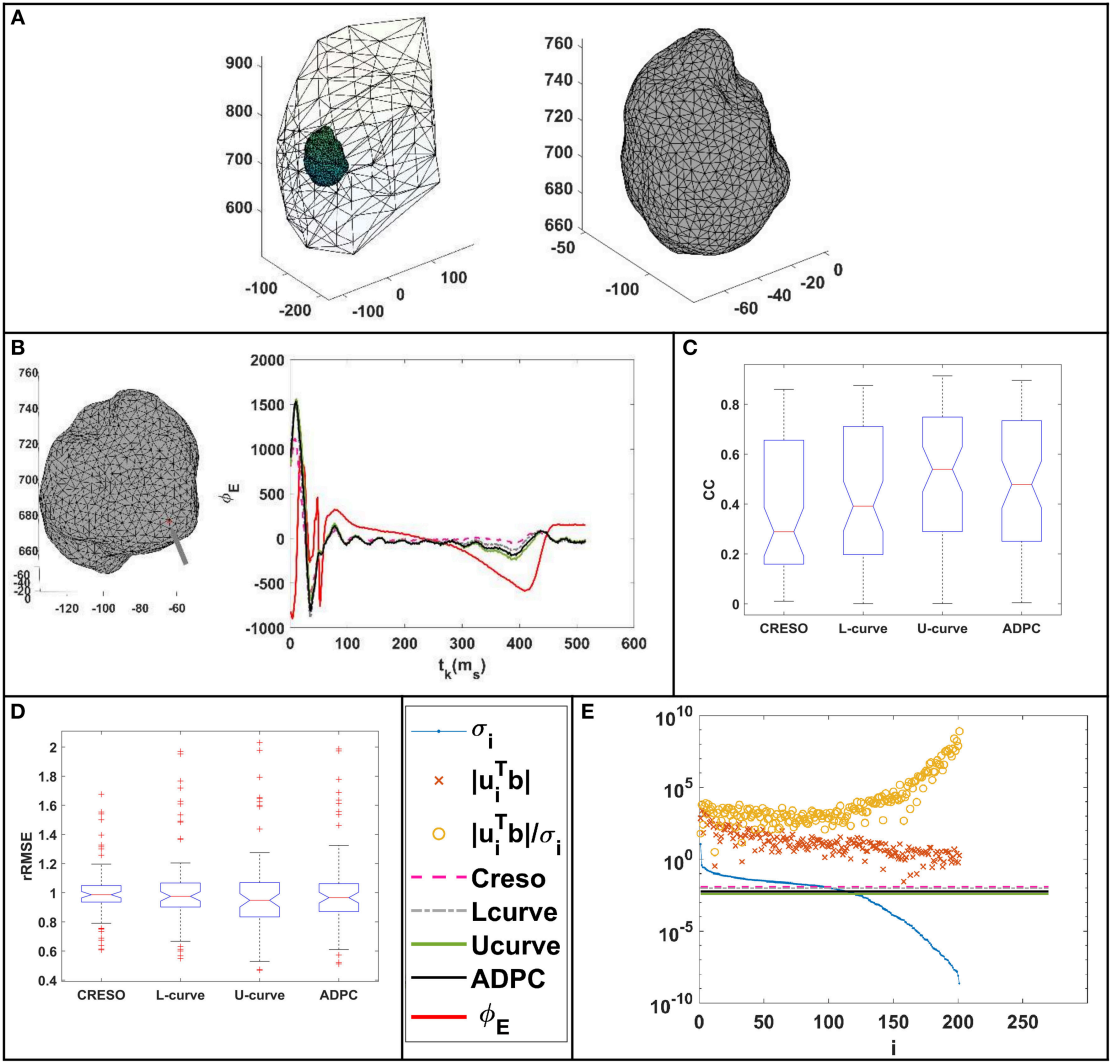
**(A)** Geometries of the canine paced heart EDGAR datasets (Cluitmans et al., 2017). **(B)** Reconstructed potentials for the different regularization parameter-choice methods against the measured ones for a point of the epicardium marked with an arrow on its geometry. **(C)** Correlation coefficients (CC) between the reconstructed potentials and the respective measured heart potentials. **(D)** Relative root-mean squared errors (rRMSE) between the reconstructed potentials and the respective measured heart potentials. **(E)** DPC plot at t_k_ = 35 ms with the different regularization parameters values holding on horizontal lines following the legend.

For the geometries of Figure 9A from the paced dog described in section Experimental data, the Figures 9B–E show: (B) the reconstructed heart potentials against the measured ones in a marked heart point, (C) the statistics boxplots of the correlation coefficients (CC), (D) the statistics boxplots relative root-mean square errors (rRMSE) and (E) the DPC plot holding the different chosen regularization values.

**Figure 10:**
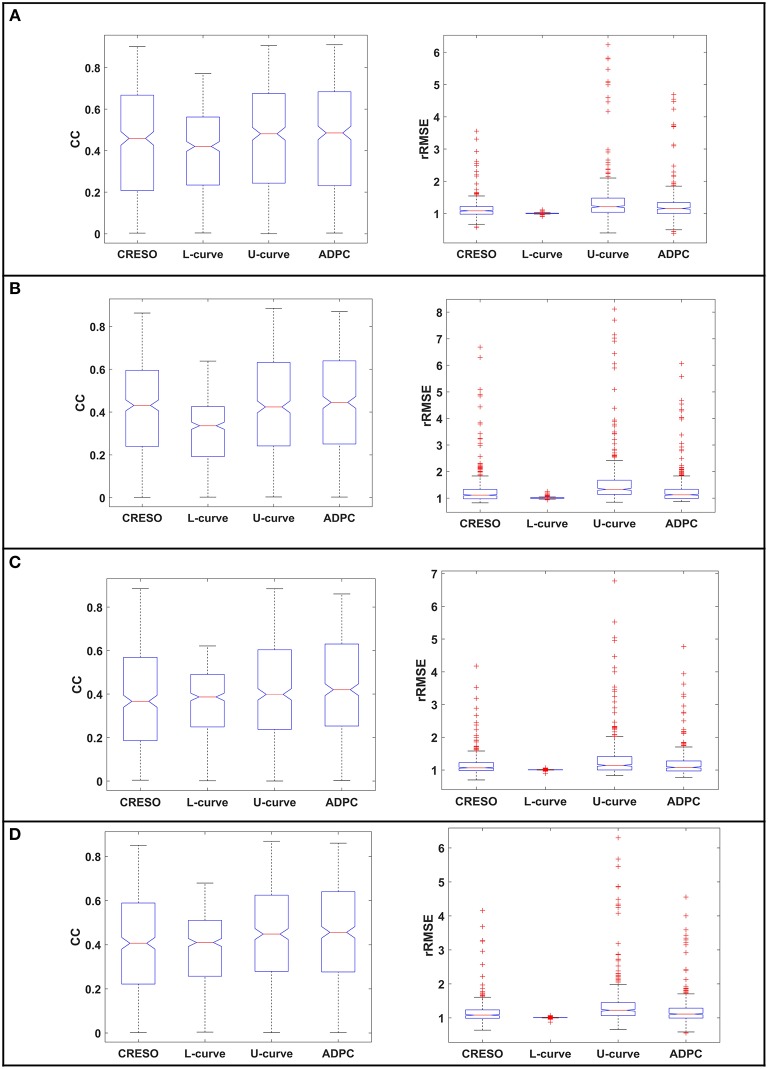
The statistics boxplot show the correlation coefficients (CC) and relative root mean-square errors (rRMSE) for the reconstructions with the different regularization parameter-choice algorithm against the respective *in-silico* heart potentials. EDGAR canine experiments: **(A)** control, **(B–D)** myocardial ischemia's (datasets number 16, 39, and 54 from the referred EDGAR experiments). The red crosses denote the outlayers.

The statistics boxplots for the different reconstructions of each paced heart and sinus rhythm datasets described in section Experimental data can be found all separately depicted in the Supplementary Material. Figure 10 also includes the separated statistic boxplots for the control and the three myocardial ischemia from a canine experiment described also in section Experimental data.

**Figure 11:**
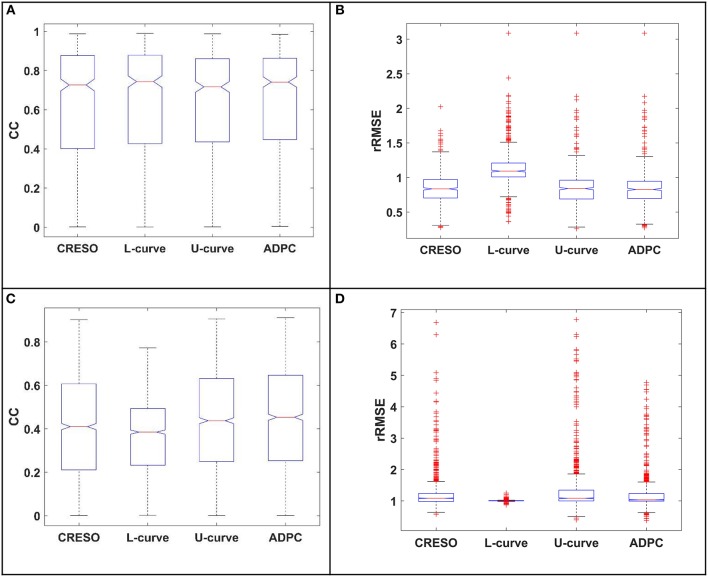
The statistics boxplots show: **(A,C)** the correlation coefficients (CC) and **(B,D)** the relative root mean-square errors (rRMSE) for the reconstructions with the different regularization parameter-choice algorithm against the respective *in-silico* heart potentials. EDGAR experiments: **(A,B)** paced and sinus rhythm dog and pig experiments together, **(C,D)** Control and myocardial ischemia datasets canine experiments from Utah together (ischemia datasets number 16, 39, and 54 from the referred EDGAR experiments) The red crosses denote the outlayers.

Finally, Figure 11 compiles the statistic boxplots for the different reconstructions of all the paced and sinus rhythm datasets together (A,B) and the control and myocardial ischemia together (C,D).

## Discussion

Two new methods were introduced to calculate the regularization parameter of the two-norm Tikhonov regularization method (referred in the manuscript as Tikhonov regularization method) when using the MFS for ECGI: The U-curve (a method never used before in cardiac applications) and the ADPC (a new automatic developed method based on DPC).

The reason for this study came about from the limitations found when using the most common parameter-choice methods (the L-Curve and the CRESO) for the ECGI MFS setting.

We focused on the introduction and validation of new automatic regularization parameter-choice methods, combining information not only about the residual norm but also about the solution norm. This choice is based on the idea of later introducing the physiologically-based prior information on the regularization term in order to improve the ECGI inverse problem, as shown in recent manuscripts (Figuera et al., 2016; Cluitmans et al., 2017; Duchateau et al., 2018; Schuler et al., 2018). To introduce the physiologically-based prior information, regularization techniques need to adjust its solution norm constraint on this information. We did not compare methods that only considered the information of the residual norm (ignoring the solution norm information), such as the cited generalized cross validation, which also did not compute a suitable regularization parameter when dealing with highly correlated errors (Hansen, 1992).

The ADPC algorithm presented here provides a suitable regularization parameter due to the behavior of the SVs of the ECGI MFS problem (decaying slower for the higher SVs and faster for the lower ones such as in Figures 3, 5, 8, 9). The fact that the ADPC parameter choice is based on the necessary DPC fulfillment for any regularization parameter for the Tikhonov regularization method (Hansen and O'Leary, 1993; Hansen, 2010) ensures an optimal solution for highly ill-posed problems. In addition, the DPC plot gives us a valuable indication of the over-regularization level of a solution. This is perfectly shown by the location of the regularization parameters in the DPC chart and the relationship of this location with their respective reconstructed potentials and the dV/dT patterns (Figures 3–6). In the first DPC plot (Figure 3F) the CRESO, the L-curve and U-curve parameters are located fairly above the moment the SVs start to decay faster, and this results in a wider QRS (losing also the S-wave in most of cases) on the respective potentials along the time plot (Figures 3A–E). The U-curve method and notably the ADPC method seem to better localize the pacing on the LV lateral midwall (Figure 4). In the *in-silico* examples included in this manuscript and the Supplementary Material, the L-curve method provided the most over-regularized solution. In the cases of the single spiral wave (Figure 5), the L-curve parameter is located even higher on the DPC plot, and it results in an extremely over-regularized reconstruction of the potentials along time (losing both, the morphology, and the amplitude of the reconstructed potentials). This therefore causes the inhibition of the computation of the corresponding dV/dT map (data not shown in Figure 6 or highlighted in Table 1 as NA^*^). Finally, regarding the dV/dT maps in Figure 6 we can clearly see the improvement of the ADPC solution against the CRESO solution. The dV/dT maps of each singular simulation dataset, reconstructed by the different methods, are included in the Supplementary Material of this manuscript.

Regarding the single site pacing simulations statistics (Figures 7A,B): (i) The correlation coefficients (CC) best center tendency is achieved by the ADPC method followed by the U-curve method. In addition, the correlation coefficients of these two methods and specially of the ADPC, have a larger upper spread out. While the ADPC has the smallest variability, it has some lower outlayers in the same range where the resulting interquartile values of other methods vary (being the interquartile the height of the boxes, 1st−3rd quartile). The outlayers indicate values greater than the 1.5 interquartile ranges away from the 25th percentiles. The L-curve solution has the worst correlation coefficients center tendency and the CRESO solution has a center tendency similar to the U-curve, but with higher variability. (ii) The relative root-mean squared errors (rRMSE) best center tendency is also achieved by the ADPC method followed by the U-curve and the CRESO method, but again the CRESO method shows a higher variability error. The L-curve solution also shows the worst performance in terms of relative root-mean squared error (lowest center tendency and highest variability). Finally, the upper outlayers from the ADPC resulting relative root-mean squared errors are located out of the other methods interquartile values. However, all these outlayers come from the *in-silico* LV lateral endocardial data as can be observed in the single simulations' boxplots of the Supplementary Material.

In the case of the single spiral simulations' statistics (Figures 7C,D): (i) The correlation coefficients (CC) best center tendency is achieved through the ADPC method. In addition, its distribution is also more focused in the upper values. However, the U-curve and the CRESO methods provide close results for correlation coefficients for the spirals than for the single site pacing simulations. Again, the ADPC has some outlayers inside the other methods' value ranges. The L-curve solution has the worst correlation coefficient center tendency and the highest variability, meaning that its performance (compared with the other methods solutions) is even worse than for the single site pacing simulations. (ii) The relative root-mean squared errors' (rRMSE) better center tendency is also achieved by the ADPC method followed by the U-curve and the CRESO methods. Here, the L-curve method has less upper outlayers but its center tendency (around 1) continues being the worst, and its correlation coefficients are higher distributed and are worse than the other methods.

In terms of the *in-silico* data dV/dT patterns statistics (Table 1): (i) In the single site pacing *in-silico* datasets, the highest correlation coefficients (CC) and lowest relative root-mean squared errors (rRMSE) are achieved by the ADPC, followed by the U-curve. The L-curve over-regularized some of the solutions that inhibit the computation of the respective activation time maps. (ii) In the case of the spirals *in-silico* datasets, the ADPC also provided the highest correlation coefficients and the lowest relative root-mean squared errors, followed by the U-curve. However, differences between the ADPC, the U-curve and the CRESO methods here are more significant in terms of correlation coefficients (morphology) than in terms of relative root-mean squared errors (amplitude), where the results are closer. Finally, the L-curve also inhibited some dV/dT map computations for the spirals *in-silico* data.

In the case of the EDGAR datasets, we found fewer differences between the different regularization parameter choice methods for the paced and sinus rhythm datasets (Figures 8, 9, 11A,B and respective separated boxplots in the Supplementary Material). However, in the case of the pig experiment described in section Experimental data (Figure 9) we could not impose compliance with the zero-flux or homogeneous Neumann conditions on the MFS solutions [such as in Wang and Rudy (2006) and the rest of the datasets of this manuscript]. This was due to some problems encountered when computing the normal directions for the geometries provided. In Figure 8D, we can see that singular values start to decay faster to zero quite late (meaning that the problem is less ill-posed than for other examples). This agrees with our previous work (Chamorro-Servent et al., 2016b) where we showed that not applying the zero flux or Neumann conditions resulted in a less ill-posed problem, less dependent on the regularization choice. Therefore, minor differences between applying different regularization parameter-choices methods were found as expected in terms of the solutions for the pig datasets. The results for these datasets are not fully comparable with the rest of the manuscript due to this change on the numerical MFS problem solved. Instead, the results of Figure 9, fully comparable in terms of correlation coefficients (CC), continues to show an improvement on the U-curve and the ADPC solutions against the CRESO and the L-curve. Nevertheless, the authors of these datasets specified in their readme file that they had a un-solved issue with the amplitude of the recorded potentials. We therefore prefer not to draw conclusions on the resulting amplitudes (relative root-mean squared error or rRMSE) for the canine paced and sinus rhythm datasets. But in terms of the morphology of potentials, the ADPC continues to be the most stable method. For the four datasets, the ADPC keeps the potentials morphology (correlation coefficients) comparable or better than the CRESO method (the gold standard) does.

Finally, referring to the control and the three myocardial ischemia datasets from the canine EDGAR experiments, the data recorded was quite noisy, as shown in the recorded potentials snapshot of the Supplementary Figure 10. This resulted in poor (very high) relative root mean square errors (rRMSE). However, this is not due to an amplitude problem of the reconstructed potentials (see the Supplementary Material) but due to the existent noise. Nevertheless, we can see an upper and better central tendency from the U-curve and the ADPC correlation coefficients (CC) compared to the other methods, when reconstructing the ischemia datasets (Figures 10B–D from manuscript). This is less appreciated in the summary of the statistics, when the control case in Figure 11C is included.

In conclusion, this study shows the feasibility of the U-curve and the ADPC techniques in the ECGI inverse problem setting, when using the MFS as a numerical method. The new techniques result in an improvement of the morphology of the reconstructed epicardial potentials and in the *in-silico* cases of their amplitude. The ADPC seems to be the most stable method to keep the morphology of potentials.

## Limitations

This study provides results for the ECGI MFS problem, such as described in Wang and Rudy (2006). The empirical lower threshold of the ADPC and the median choice works well due to the behavior of the decay of the singular values of the MFS matrix (see Figures 3F, 5F, 8D, 9E). However, it is well-known that parameter-choice methods are problem dependent (Hansen, 2010). Note for example that the authors in Milanič et al. (2014), Cluitmans et al. (2015), Figuera et al. (2016) found suitable results through the L-curve method when using the BEM as a numerical model, which is not always the case when using the MFS instead.

As explained in the discussion, we focused on automatic methods that can be extended to include physiologically-based prior information. Nevertheless, for the cases where physiologically-based prior information of the solution could not be provided, it can be interesting to compare our methods with the generalized cross validation method.

A finer discretization of the AT for visualization, would be more sensible and provide more continuous data. In addition to improving the AT maps accuracy, methods such the one described in Duchateau et al. (2017) can be used.

While we anticipate in section U-curve that the U-curve method is computationally cheaper than the L-curve (due to its prior interval) (Krawzyck-Stando and Rudnicki, 2007; Chamorro-Servent et al., 2011; Chen et al., 2016), we need further studies, in terms of the computational burden of the whole parameter choice method.

If anyone wanted to use the new ADPC or the U-curve method, with other numerical problems such as the BEM, the FEM or even the MFS with different placement of the virtual source points such as (Chamorro-Servent et al., 2016a), or different boundary conditions such as (Chamorro-Servent et al., 2016b), we recommend repeating this study before drawing further conclusions. A clear example of this is shown with the results from the pig experiments (Figures 8, 11A,B), where we did not impose to the solution compliance with the zero-flux or homogeneous boundary conditions, and we found fewer differences between the methods, in agreement with (Chamorro-Servent et al., 2016b).

Finally, the ADPC method and the L-curve based on the mathematical solution of a problem with l2-norm constraints (Hansen, 2010) such as the one presented here, and may not perform as well when using constraints based on another norm (for example the l1-regularization norm) (Hansen, 2010). If l1-norm prior-information needs to be added, then the ADPC method will not work because it is based on the DPC. PC Hansen, the author of DPC (Hansen, 1990, 2010) has explained this issue well in his work. The poor performance of ADPC or L-curve in l1-regularization approaches is not due to a lack of robustness of the DPC or the method, but due to a misusage. The mathematical basis of both, the condition and the method, is the l2-norm Tikhonov solution definition. The DPC is a condition that must fulfill any regularization parameter for the l2-norm Tikhonov approach. In the latter, i.e., cases involving other regularization norm terms, the U-curve method may provide better results.

## Outlook

This study assumed that no *a priori* physiologically information about the epicardial potentials were available, while studying regularization parameter-choice methods that can be adjusted to problems introducing different l2-norm constraints. Due to the increasing number of work that proposes the incorporation electrophysiological knowledge (Figuera et al., 2016; Cluitmans et al., 2017; Duchateau et al., 2018; Schuler et al., 2018), it would be interesting to see how the U-curve and ADPC adapted methods perform when including electrophysiological prior knowledge into a l2-norm constraint.

The reader may observe that the ADPC and the U-curve continued to preserve the morphology for experimental data and specifically for high noisy data, such as the control and the three myocardial ischemia datasets from the canine EDGAR experiments (Figure 10 and Supplementary Figure 10). However, it will be interesting to develop a noise robustness study for the *in-silico* data, both including noise on the measured datasets and on the geometric locations of the electrodes.

## Ethics Statement

The experimental data used is from EDGAR.

## Author Contributions

JC-S designed and developed the ADPC method, and developed the regularization criteria techniques and the MFS, designed and conducted the study and wrote the manuscript. RD and YC provided technical expertise and contributed during manuscript preparation.

### Conflict of Interest Statement

The authors declare that the research was conducted in the absence of any commercial or financial relationships that could be construed as a potential conflict of interest.
