# Supplementary material for: Considering New Regularization Parameter-Choice Techniques for the Tikhonov Method to Improve the Accuracy of Electrocardiographic Imaging
Source: Front Physiol. 2019 Mar 27;10:273. doi: 10.3389/fphys.2019.00273 (PMC6445955; doi:10.3389/fphys.2019.00273)
Supplement: Supplementary file 1 [file Data_Sheet_1.pdf]

Supplementary Material

1 Supplementary Figures and Tables

We included here supplementary figures about the in-silico and the experimental data used in the manuscript.

For the in-silico data, we reconstructed eight datasets (four single pacing site and four spirals waves). However, we only showed: i) the dV/dT patterns of one single pacing site and one spiral datasets result; ii) the two summaries of the statistics (one for all the single pacing datasets results together and the other for the spiral datasets results). Then, we decided to include in this supplementary material the dV/dT maps of each remaining singular dataset (**Supplementary Figures 1-6**). In addition, we included the statistics study (boxplots of the correlations coefficients and the relative root-mean square errors) of each of the eight in-silico datasets separately (**Supplementary Figures 7-8**).

For the experimental data, we included the statistics study of each dataset separately of the control and the myocardial ischemia canine experiment (**Supplementary Figure 9**), the snapshots showing the differences between the reconstructed epicardial potentials for each regularization parameter method (**Supplementary Figure 10**), as well as their respective epicardial and body surface potentials.

1.1 Supplementary Figures for the in-silico data

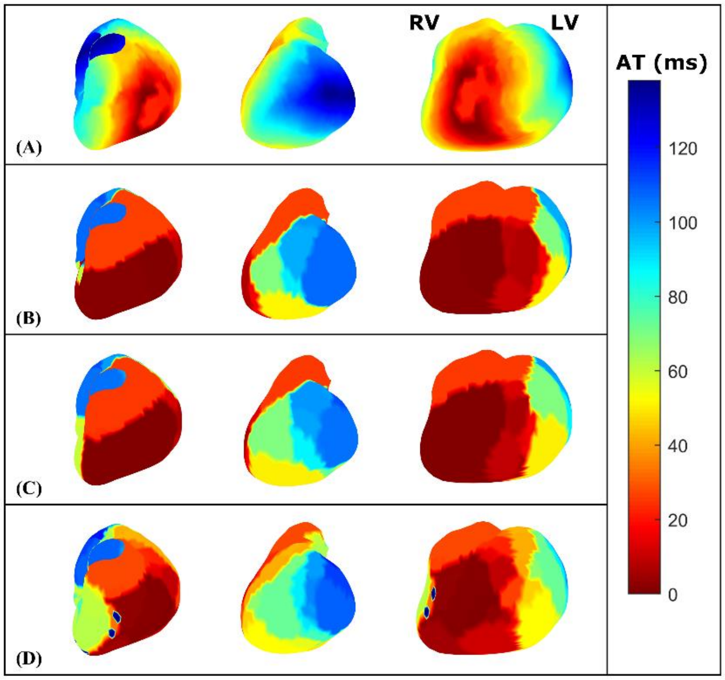

Supplementary Figure 1. AT maps of the single pacing right ventricle free wall site in-silico dataset: (A) Reference or in-silico, (B) CRESO, (C) U-curve, (D) ADPC. The L-curve solution is not shown due to the inhibition of its AT map.

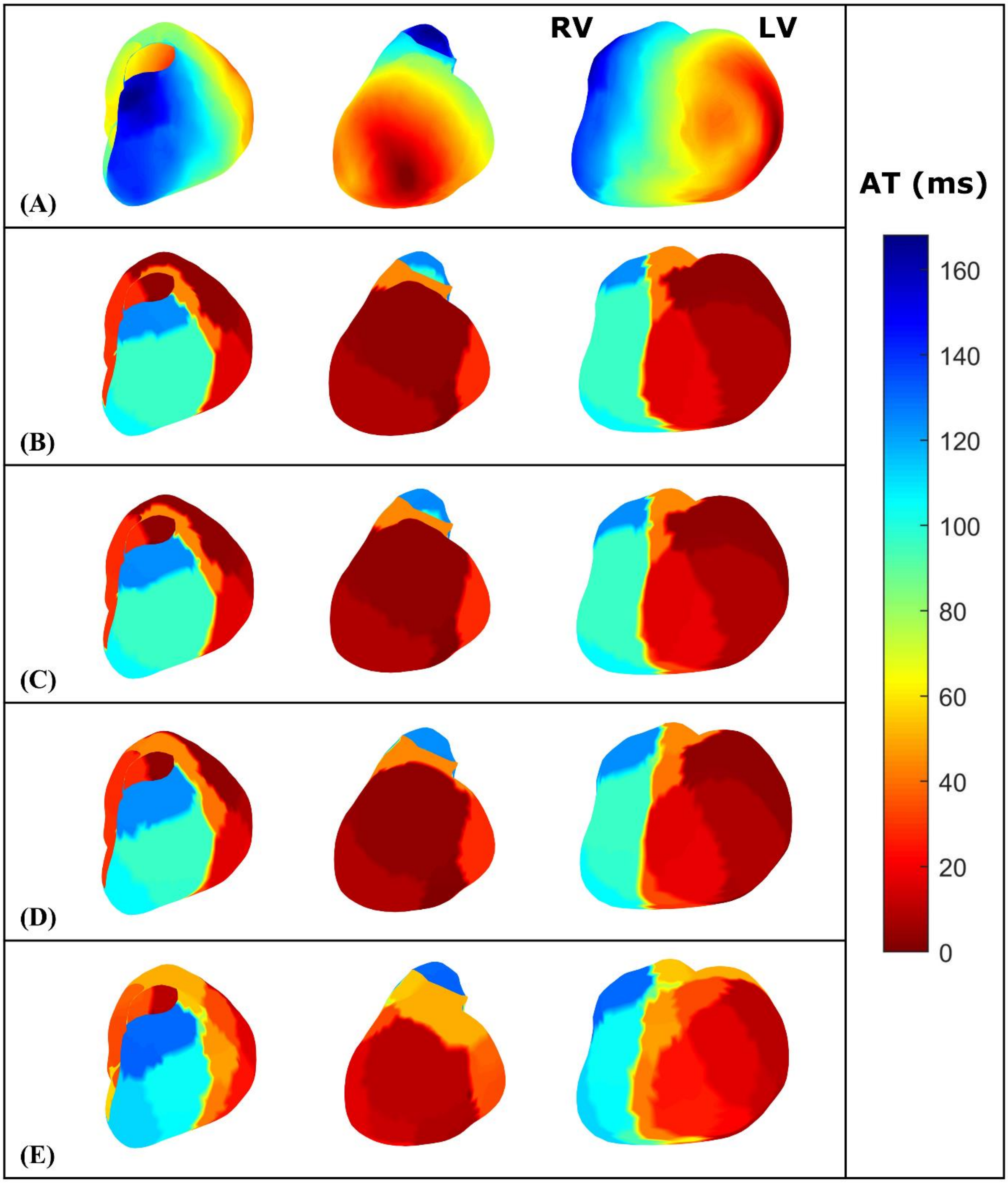

Supplementary Figure 2. AT maps of the single pacing left ventricle lateral endocardial site in-silico dataset: (A) Reference or in-silico, (B) CRESO, (C) L-curve, (D) U-curve, (E) ADPC.

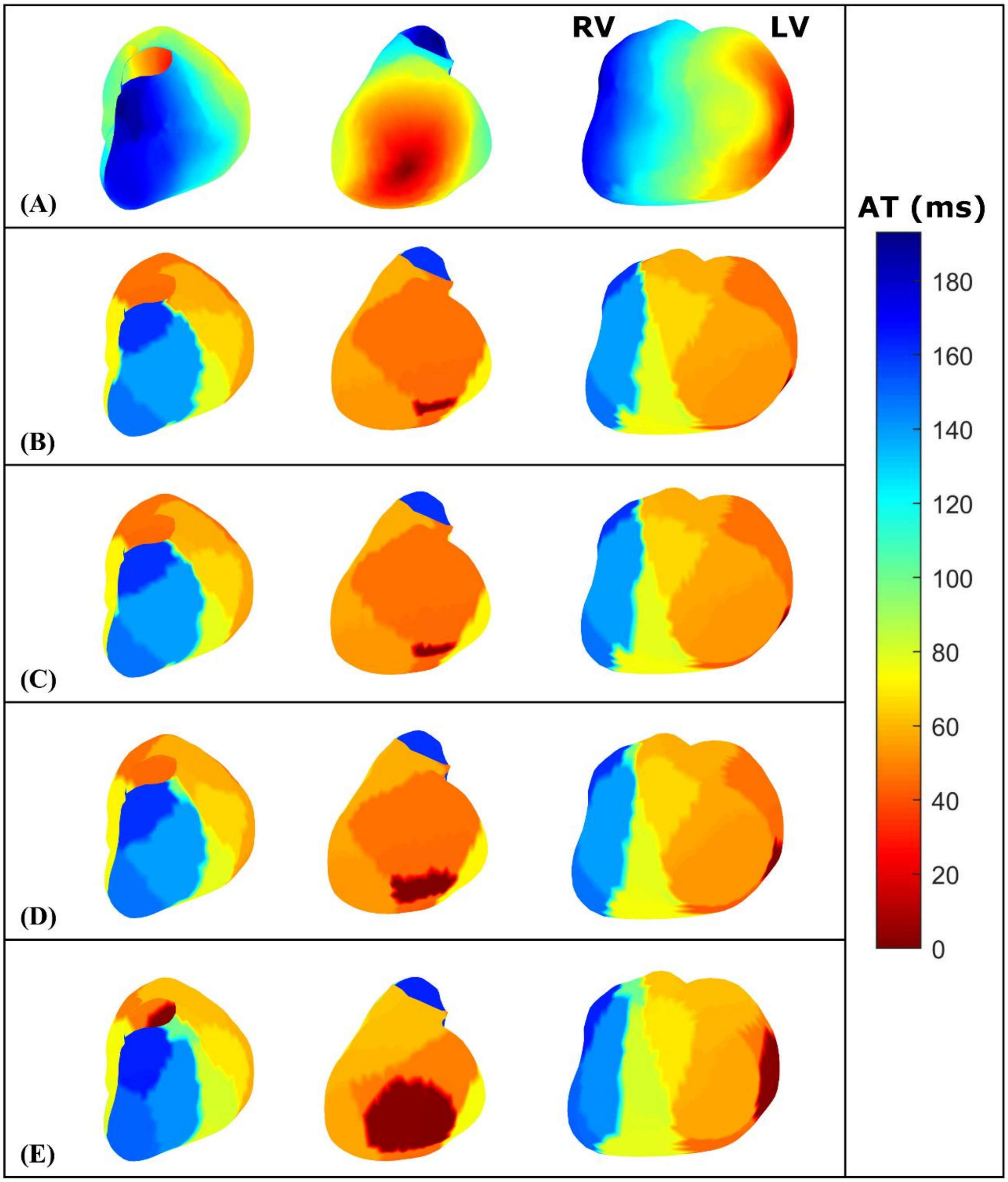

Supplementary Figure 3. AT maps of a single pacing left ventricle lateral midwall site in-silico dataset: (A) Reference or in-silico, (B) CRESO, (C) L-curve, (D) U-curve, (E) ADPC.

The results of the **Supplementary Figures 1-3** show that the ADPC kept better the morphology of the AT maps than the other methods. The results for the pacing on the left ventricle lateral endocardial dataset were poor (**Supplementary Figure 2**), but still better than the results provided by the other regularization parameter-choice methods. This can also be observed on the boxplots of the **Supplementary Figure 7** in terms of correlations coefficients and relative root mean square errors between each regularization parameter-choice reconstructed heart potentials and the respective in-

silico ones. Finally, the L-curve inhibited the computation of the AT map for the single beat pacing on the right ventricle free wall.

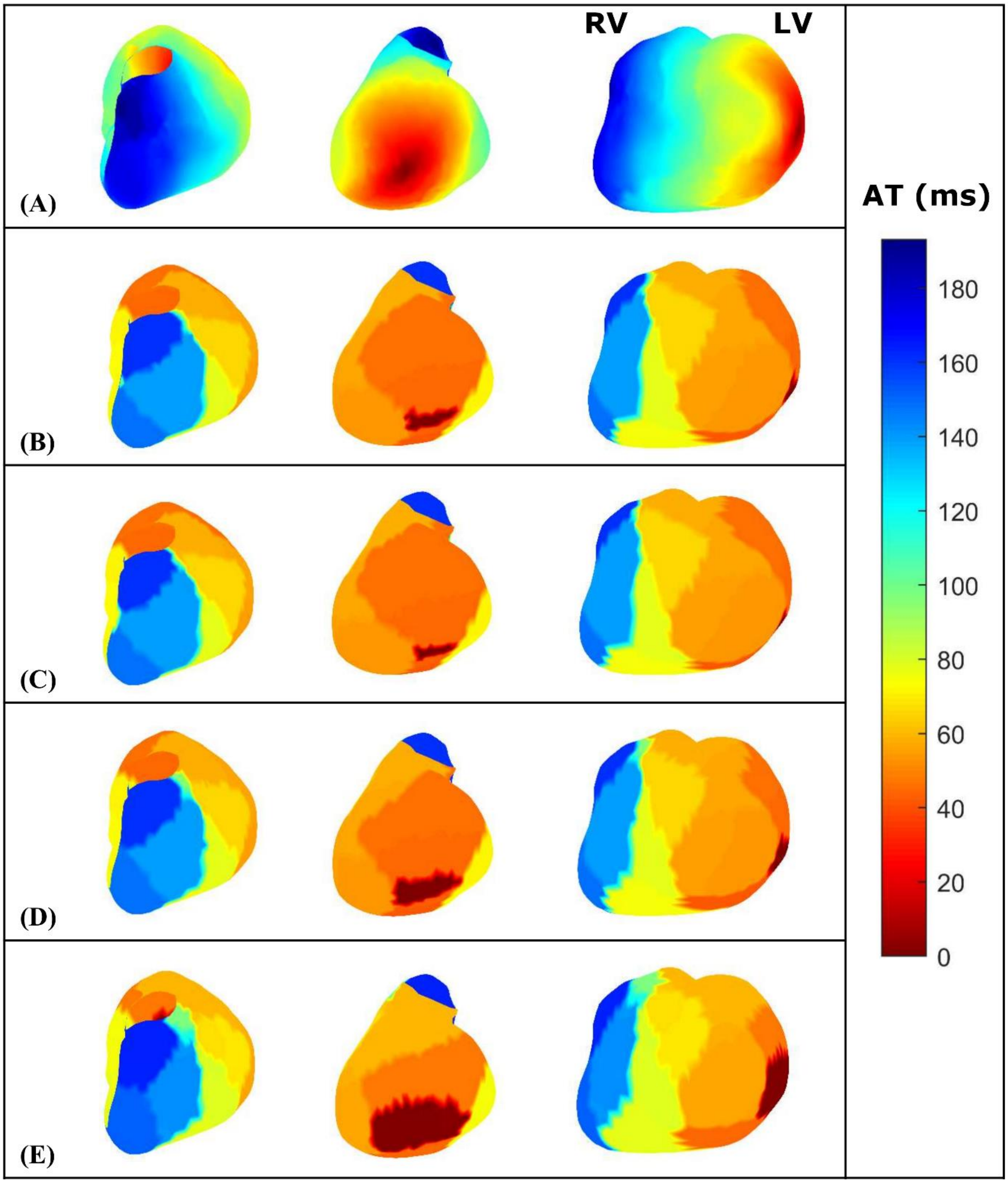

Supplementary Figure 4. dV/dT patterns of a single spiral wave and break in-silico dataset: (A) Reference or in-silico, (B) CRESO, (C) L-curve, (D) U-curve, (E) ADPC.

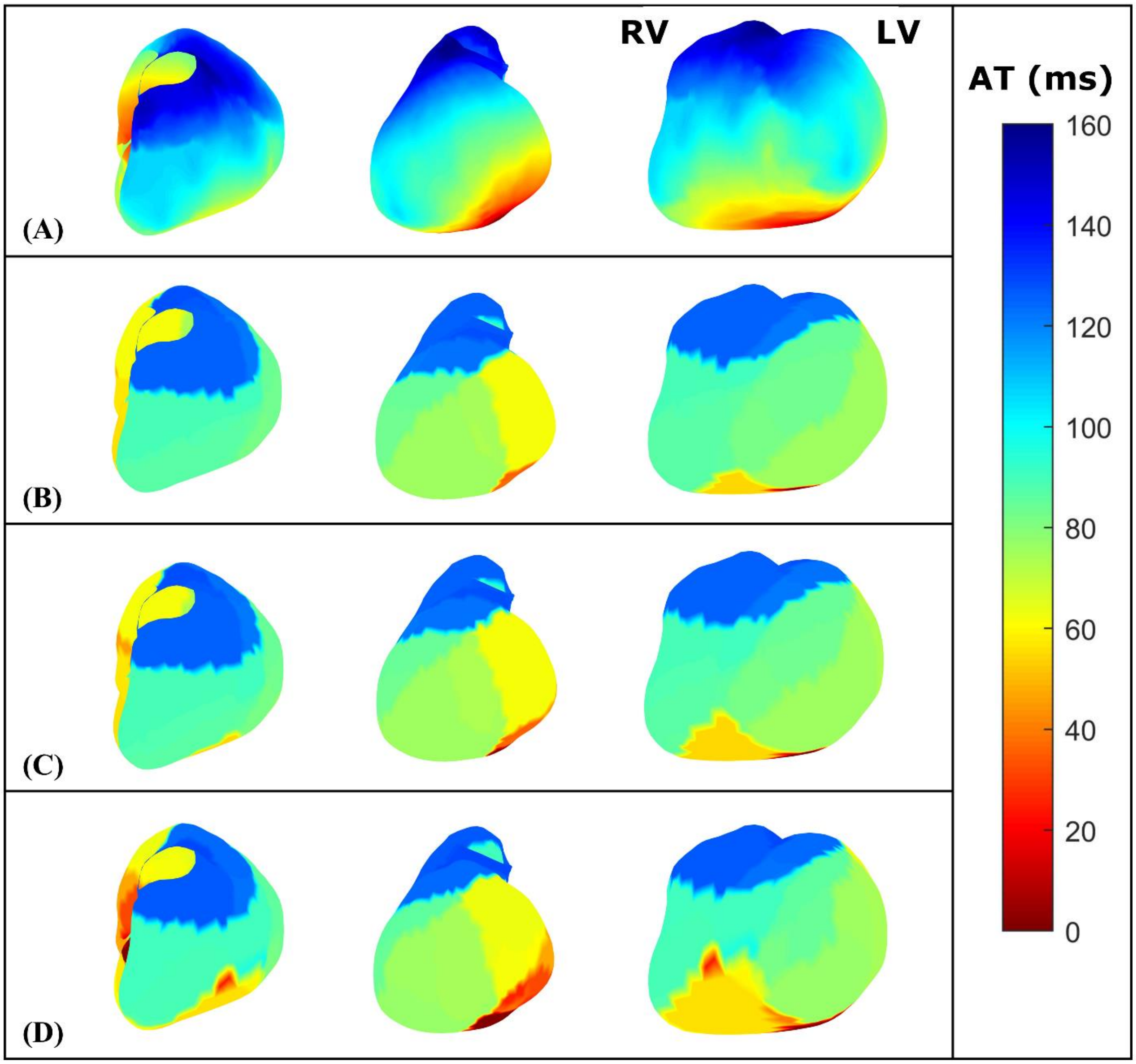

Supplementary Figure 5. dV/dT patterns of a single spiral wave in-silico dataset: (A) Reference or in-silico, (B) CRESO, (C) U-curve, (D) ADPC.

The L-curve inhibited the computation of the dV/dT patterns for almost all the spiral simulated data (**Supplementary Figures 4-6**), except for the spiral wave and break in-silico dataset used in the **Supplementary Figure 4**. Again, between the different regularization parameter-choice methods used here, the ADPC is the method that most kept the dV/dT patterns morphology. However, for the spiral in-silico cases there are minor differences between the U-curve solutions and the CRESO ones. Similarly, these results can be also appreciated in the boxplots of **Supplementary Figure 8** in terms of correlations coefficients and relative root mean square errors between each regularization parameter-choice reconstructed heart potentials and the respective in-silico ones.

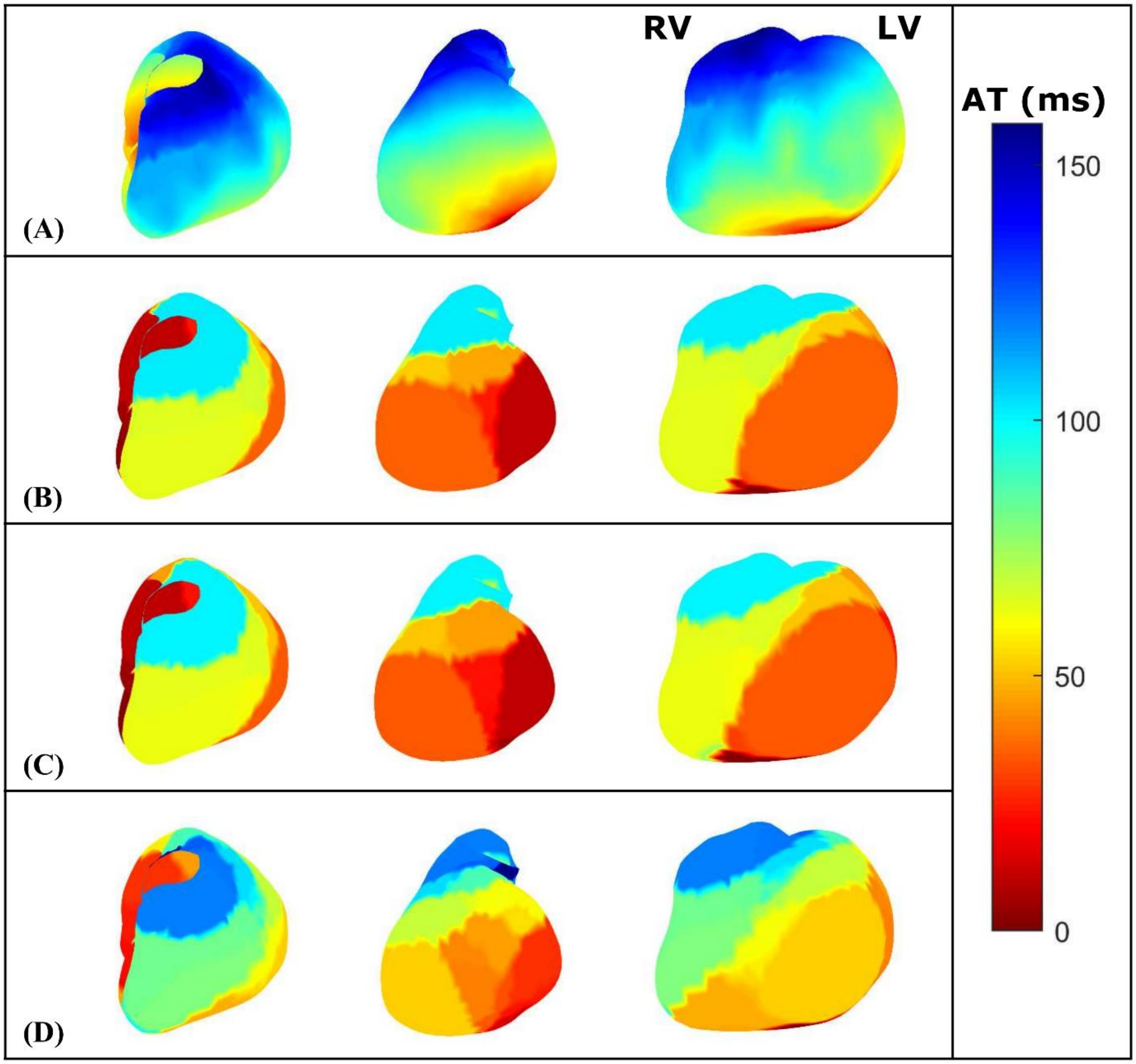

Supplementary Figure 6. dV/dT patterns of a single spiral wave with increased transmural conductivity in-silico dataset: (A) Reference or in-silico, (B) CRESO, (C) U-curve, (D) ADPC.

In all the single site in-silico datasets, the ADPC method (followed by the U-curve one) provided the best center tendency and the lowest variability in terms of correlation coefficients (CC) and relative root-mean square errors (rRMSE). In addition, we can clearly see that the ADPC only showed significant outlayers for the pacing site on the left ventricle lateral endocardial in-silico dataset. These significant outlayers on the relative root mean-squared errors are the ones appeared on the summarized boxplots (when compiling the statistics from all the in-silico single pacing datasets) showed on subplots B and D of **Figure 7** of the manuscript. This agrees with the poor AT map recovered in the **Supplementary Figure 1** for this pacing site in-silico dataset. It means

that the global upper outlayers appeared on the summary relative root-mean square errors (rRMSE) statistics **Figure 7B,D** from the manuscript came all from this case.

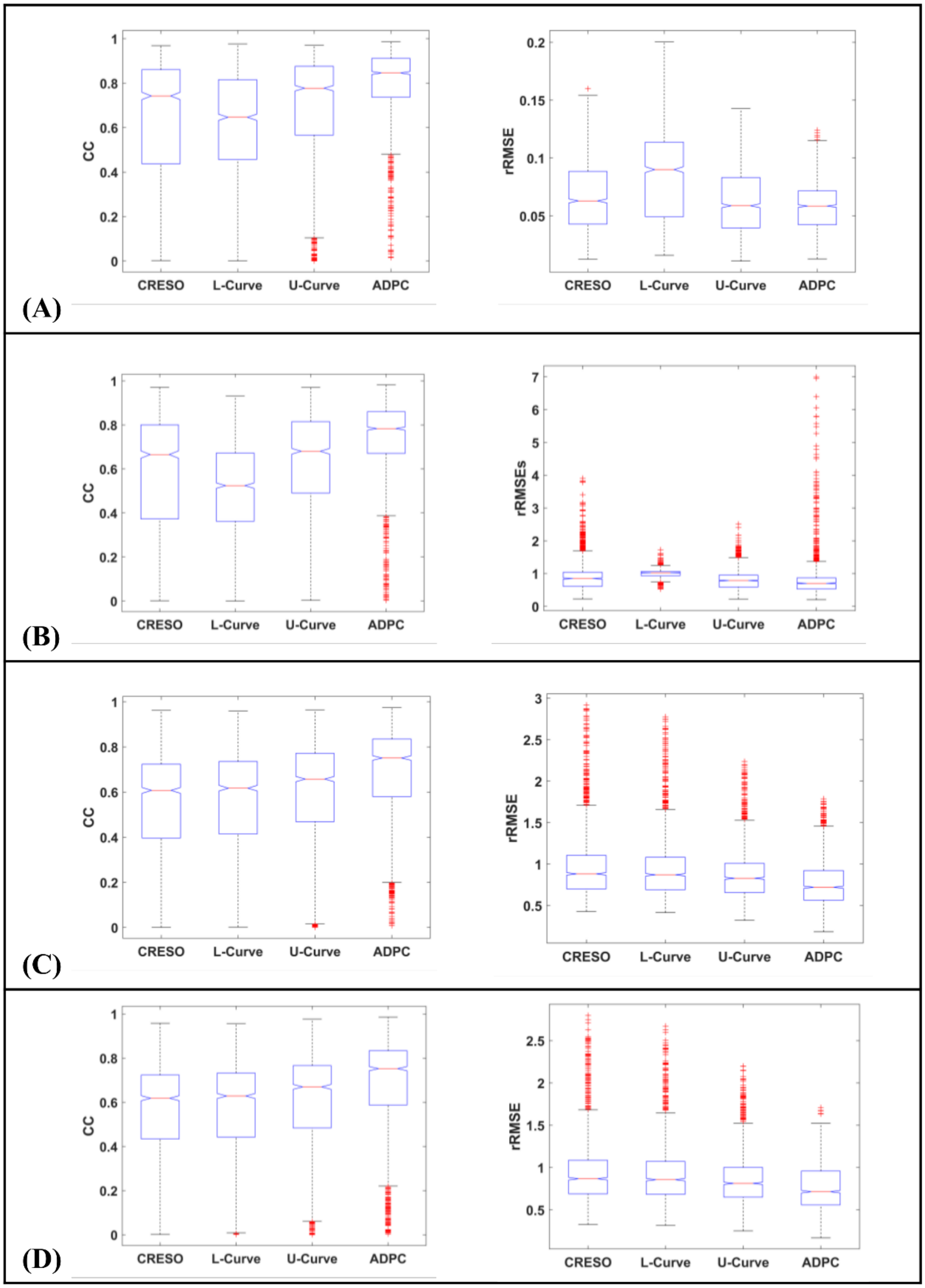

Supplementary Figure 7: The statistics show the correlation coefficients (CC) and the relative root mean-square errors (rRMSE) boxplots for the reconstructions with the different regularization

parameter-choice algorithm against the respective in-silico heart potentials. The red crosses represent the outlayers. Single pacing site in-silico datasets studied: (A) Right Ventricle free wall, (B) Left Ventricle lateral endocardial, (C) Left Ventricle lateral midwall, (D) Left Ventricle lateral epi.

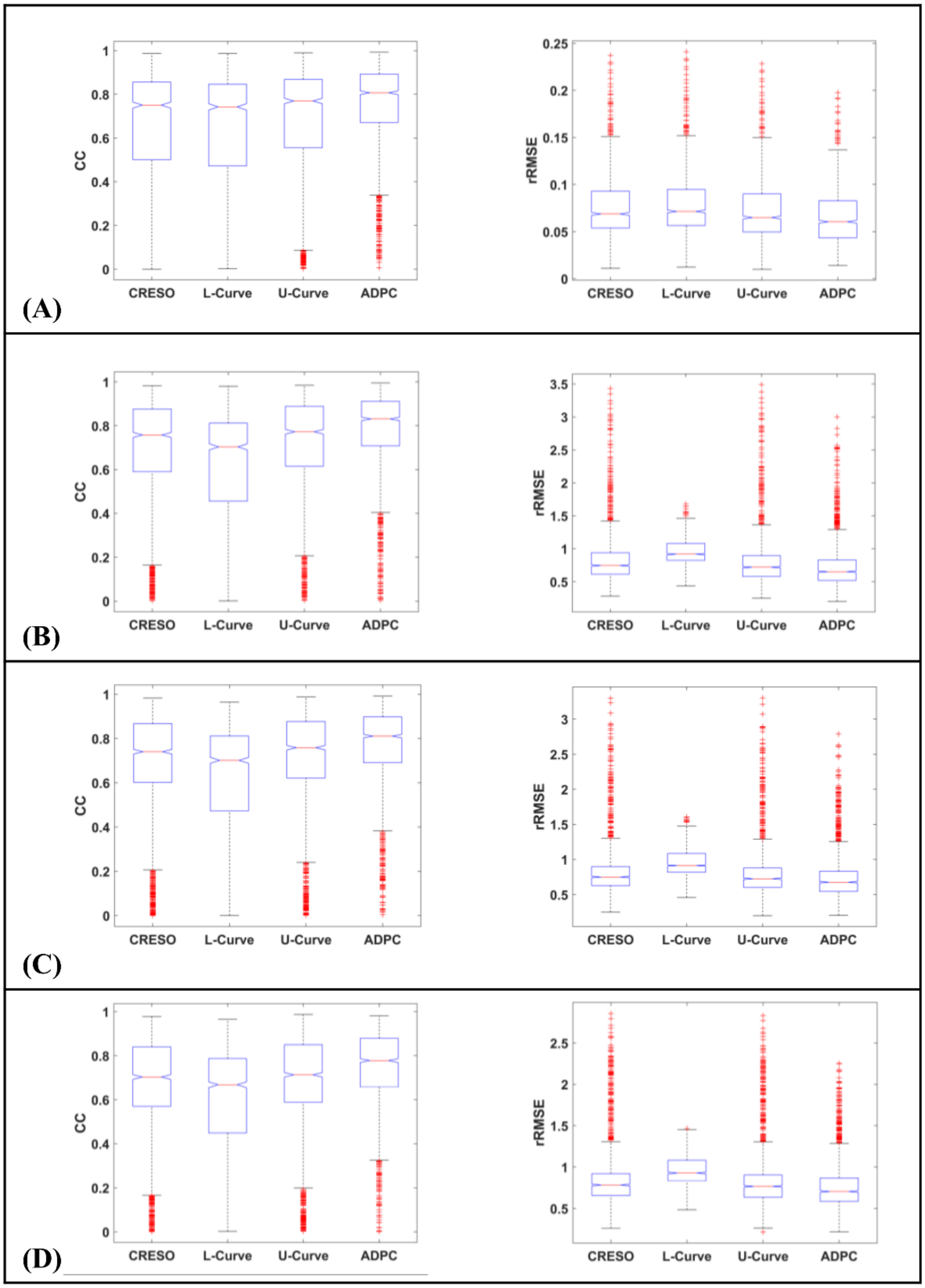

Supplementary Figure 8: The statistics show the correlation coefficients (CC) and the relative root mean-square errors (rRMSE) boxplots for the reconstructions with the different regularization parameter-choice algorithm against the respective in-silico heart potentials. The red crosses represent

the outlayers. Spiral in-silico dataset studied: (A) Single spiral wave and break, (B) Single spiral wave, (C) Single spiral wave with increased transverse conductivity, (D) Single spiral wave with increased transmural conductivity.

The ADPC method provided the best center tendency and the lowest variability in terms of correlation coefficients (CC) and relative root-mean square errors (rRMSE) for all the spiral in-silico datasets. However, while the U-curve improved the center tendency and the variability of the correlation coefficients and the relative root-mean square error for the in-silico single pacing datasets against the CRESO solutions; this was not the case for the in-silico spiral datasets (where both methods did not offer major differences between them).

1.2 Supplementary Figures for the experimental data

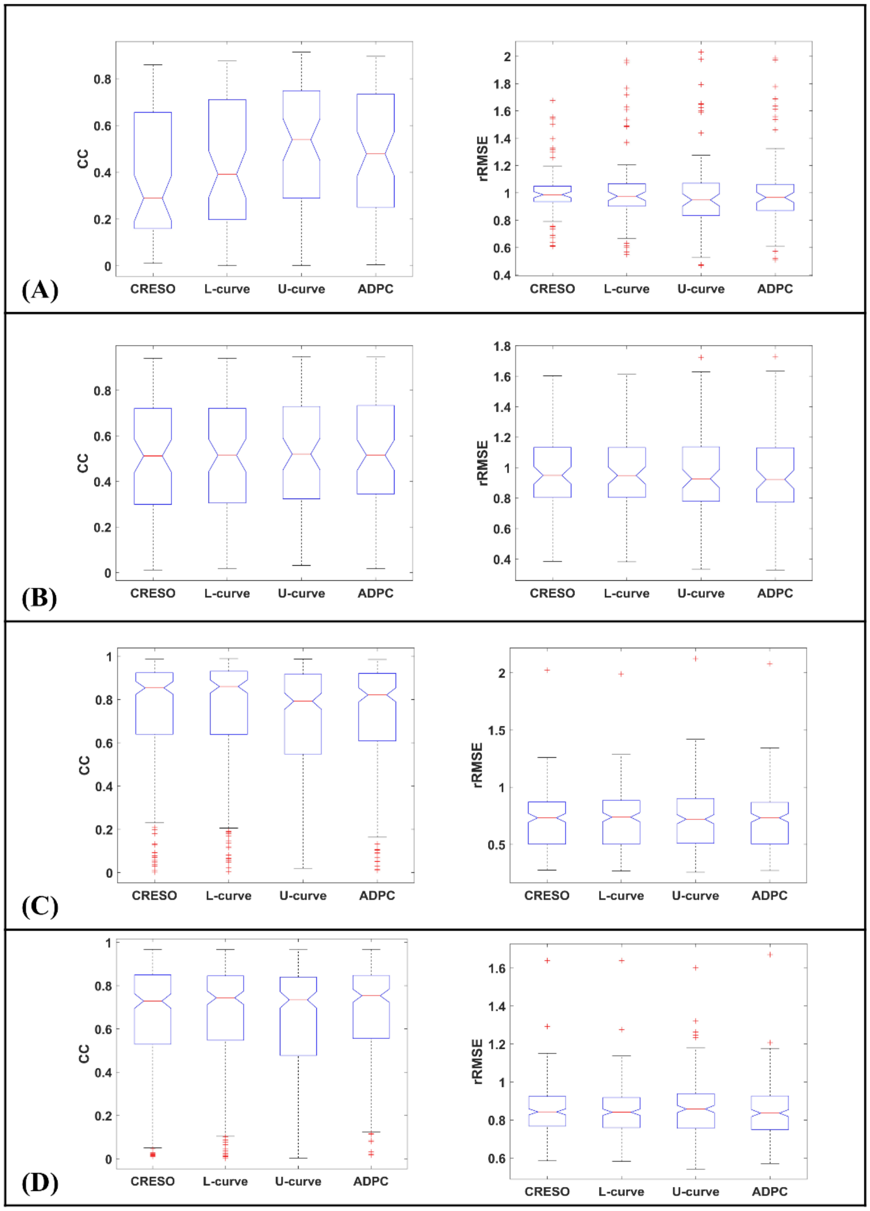

Supplementary Figure 9: Boxplots for everyone paced or sinus rhythm experimental dataset. The statistics show the correlation coefficients (CC) and the relative root mean-square errors (rRMSE)

boxplots for the reconstructions with the different regularization parameter-choice algorithm against the respective in-silico heart potentials. The red crosses represent the outlayers. Spiral in-silico dataset studied. Dog EDGAR experiments from Maastricht: (A) paced, (B) sinus rhythm. Pig EDGAR experiments from Auckland: (C) paced, (D) sinus rhythm**.**

For these experiments the improvement of the ADPC is less notably, and the U-curve shows greater variability on the correlation coefficients distributions. However, in the case of the paced dog experiment we continue to have a better center tendency of the correlation coefficients, and an upper values tendency for the U-curve and the ADPC solutions compared to the other methods. We have also some upper tendency correlation coefficients for both sinus rhythm data (**Supplementary Figure 9 C,D)** when using the ADPC, but this difference is lower notably than it was for the in-silico datasets presented before or the ischemia datasets from **Figure 10** of the manuscript. Nevertheless, as concluded in the manuscript, if we must choose a method keeping the potentials morphology, we will continue choosing the ADPC method due to its less variability on the correlation coefficients between the different datasets. The CRESO method for example shows quite nice results for the pig paced dataset and quite poor results for the paced dog one. For this data we have better results than before regarding the L-curve method.

As described in the paper, in the case of the EDGAR pig datasets used here we did not apply the zero flux or homogeneous Neumann conditions on the MFS solutions due to some problems computing the normal directions for this case. In the **Figure 8D** from the manuscript we can see that the singular values start to decay to zero quite late (meaning that the problem is better posed than for other examples). In agreement with our previous work [17], minor difference was found between applying different regularization parameter-choice methods for these datasets.

Regarding the amplitude of the reconstructed potentials or the relative root mean square error (rRMSE) from the dog datasets reconstructions, we can see only a better central tendency on the dog sinus rhythm dataset (**Supplementary Figure 9B**). However, the authors of these datasets specified in the readme file that they had a non-solved issue with the amplitude of the recorded potentials. Then, we prefer to do not take conclusions on the resulted amplitudes here.

The canine myocardial ischemia EDGAR dataset was quite noisy, as well as its torso data as shown in the recorded potentials snapshots from **Supplementary Figure 10**. This resulted in poor relative mean square errors (as shown in the manuscript). However, in the reconstructed potentials below it can be seen that this is due to the noise and not to a high amplitude error. However, the U-curve and the ADPC continue to keep the morphology of the potentials.

The L-curve highly over-regularized the solution for the control and for the three myocardial ischemia of the canine experiments, losing completely the QRST morphology (**Supplementary Figure 10**). However, for these examples, seems that the U-curve and the ADPC resulted on noisy reconstructed potentials while improving the QRST morphology and not losing amplitude against the CRESO reconstructed potentials.

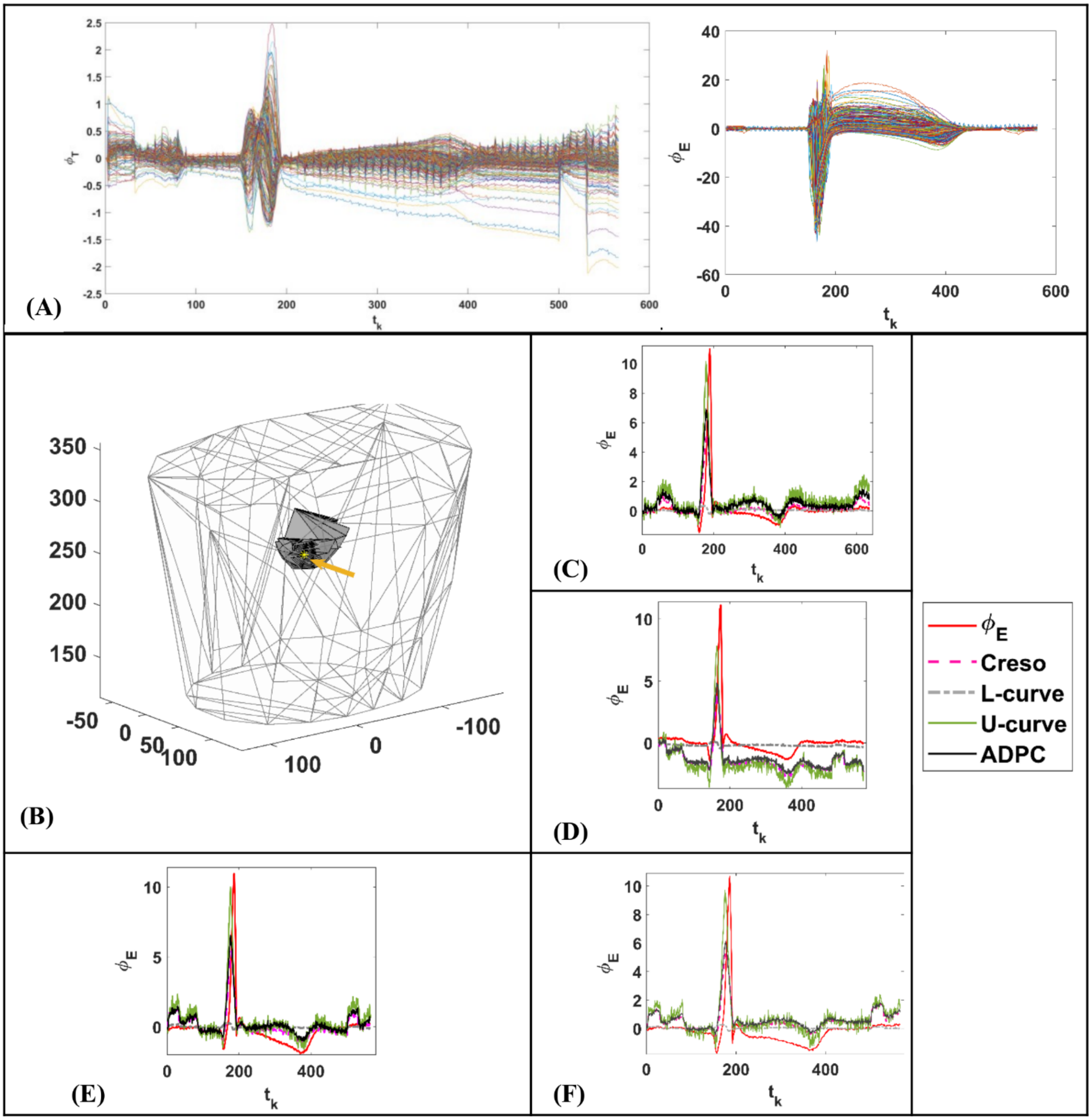

Supplementary Figure 10: (A) Recorded torso potentials and heart potentials for the myocardial ischemia case solved on E. (B) Torso and heart sock electrodes geometries from the canine experiment. (C-F) Reconstructed potentials against measured ones (in the sock location showed in B with yellow arrow and star) for: (C) a control and (D-F) three myocardial ischemia. This figure refers to the solution for the canine experiments from Utah together (ischemia datasets number 16, 39 and 54 from the referred EDGAR experiments).
